# Cryo-EM structure of the four-subunit *Rhodobacter sphaeroides* cytochrome *bc*_1_ complex in styrene maleic acid nanodiscs

**DOI:** 10.1073/pnas.2217922120

**Published:** 2023-03-13

**Authors:** David J. K. Swainsbury, Frederick R. Hawkings, Elizabeth C. Martin, Sabina Musiał, Jack H. Salisbury, Philip J. Jackson, David A. Farmer, Matthew P. Johnson, C. Alistair Siebert, Andrew Hitchcock, C. Neil Hunter

**Affiliations:** ^a^School of Biological Sciences, University of East Anglia, Norwich NR4 7TJ, United Kingdom; ^b^Plants, Photosynthesis and Soil, School of Biosciences, University of Sheffield, Sheffield S10 2TN, United Kingdom; ^c^Diamond Light Source Ltd, Didcot OX11 0DE, United Kingdom

**Keywords:** cytochrome *bc*_1_, quinone, *Rhodobacter sphaeroides*, evolution, photosynthesis

## Abstract

Cytochrome *bc*_1_ complexes are ubiquinol:cytochrome *c* oxidoreductases, and as such, they are centrally important components of respiratory and photosynthetic electron transfer chains in many species of bacteria and in mitochondria. The minimal complex has three catalytic components, which are cytochrome *b*, cytochrome *c*_1_, and the Rieske iron–sulfur subunit, but the function of mitochondrial cytochrome *bc*_1_ complexes is modified by up to eight supernumerary subunits. The cytochrome *bc*_1_ complex from the purple phototrophic bacterium *Rhodobacter* *sphaeroides* has a single supernumerary subunit called subunit IV, which is absent from current structures of the complex. In this work we use the styrene–maleic acid copolymer to purify the *R. sphaeroides* cytochrome *bc*_1_ complex in native lipid nanodiscs, which retains the labile subunit IV, annular lipids, and natively bound quinones. The catalytic activity of the four-subunit cytochrome *bc*_1_ complex is threefold higher than that of the complex lacking subunit IV. To understand the role of subunit IV, we determined the structure of the four-subunit complex at 2.9 Å using single particle cryogenic electron microscopy. The structure shows the position of the transmembrane domain of subunit IV, which lies across the transmembrane helices of the Rieske and cytochrome *c*_1_ subunits. We observe a quinone at the Q_o_ quinone-binding site and show that occupancy of this site is linked to conformational changes in the Rieske head domain during catalysis. Twelve lipids were structurally resolved, making contacts with the Rieske and cytochrome *b* subunits, with some spanning both of the two monomers that make up the dimeric complex.

## Significance

Cytochrome *bc*_1_ complexes are found in mitochondria and many species of bacteria and are related to the cytochrome *b*_6_*f* complexes of plants, algae, and cyanobacteria. The simplest cytochrome *bc*_1_ complexes are comprised of three core proteins, but many have acquired supernumerary subunits during evolution. The cytochrome *bc*_1_ complex of *Rhodobacter sphaeroides* has a unique supernumerary subunit, named subunit IV, which enhances its activity. We have determined the structure of the four-subunit cytochrome *bc*_1_ complex from this model phototroph, which provides insight into how and why cytochrome *bc*_1_ complexes evolved supernumerary subunits. Our structure also reveals the position of bound lipids and rationale for how the binding of quinones to the catalytic sites may coordinate the conformational changes that underpin the Q-cycle.

Cytochrome (cyt) *bc*_1_ complexes are quinol:cytochrome *c* oxidoreductases (E.C. 1.10.2.2) and are central components of respiratory and photosynthetic electron transport chains in bacteria and mitochondria. These multisubunit complexes translocate protons across bioenergetic membranes, generating a proton-motive force (pmf) that can be used to directly power cellular functions (e.g., flagella rotation and substrate transport) or stored in a chemical form as adenosine triphosphate (ATP). Similar to the related cytochrome *b*_6_*f* complex (cyt *b*_6_*f*) found in chloroplasts and cyanobacteria, cyt *bc*_1_ complexes operate via a modified Q-cycle, in which oxidation of two quinols at the Q_o_ site (also known as Q_P_) leads to the release of four protons on the positive side of the membrane, the reduction of soluble electron carriers in the lumen/intermembrane space, and the regeneration of a quinol at the Q_i_ site (also known as Q_n_) coupled to the uptake of two protons from the cytoplasm/matrix (1, 2). This bifurcated electron transfer mechanism increases the number of protons translocated per quinol oxidized and explains the high coupling efficiency of cyt *bc*_1_ and cyt *b*_6_*f* complexes. Quinol oxidation by these complexes is the rate-limiting step in photosynthetic electron transport, further underlying the central importance of cyt *bc*_1_ and cyt *b*_6_*f* in electron transfer chains (3–11).

The simplest cyt *bc*_1_ complexes, such as the bacterial complexes from *Rhodobacter* *capsulatus* or *Paracoccus denitrificans,* comprise three subunits: cyt *b*, which binds two *b*-type hemes (*b*_L_ and *b*_H_) one quinol and one quinone; cyt *c*_1_ that binds one *c*-type heme; and the Rieske subunit that binds a two-iron two-sulfur cluster (FeS). The two heme-binding domains are static during the Q-cycle, whereas the membrane-extrinsic head of the Rieske subunit is mobile and undergoes a conformational change during turnover (12, 13). These three catalytic subunits associate to form a dimer with C2 symmetry, and are common to all Rieske/cyt *b* complexes, but the mitochondrial cyt *bc*_1_ complexes (known as complex III) have a varying number of supernumerary subunits, of which there are seven in yeast and plants and eight in mammals (14). In thermophilic bacteria such as *Aquifex aeolicus,* the cyt *c*_1_ subunit has an additional N-terminal transmembrane helix (TMH) that provides a fused supernumerary component of the complex (15). It is unclear why supernumerary components were recruited during evolution, given that three subunits are sufficient for function.

*Rhodobacter (*R*.) sphaeroides* belongs to the α-proteobacteria, the class of bacteria thought to be the endosymbiotic progenitors of the mitochondria (16). Illuminating photosynthetic reaction centers (RCs) in native chromatophore membranes from this phototrophic bacterium elicits turnover of cyt *bc*_1_ complexes, providing a level of control that was instrumental for understanding the mechanism of cyt *bc*_1_ and for developing and refining the Q-cycle model; examples include refs. 1 and 17–23, and also a more recent review, ref. 24. Early preparations of this cyt *bc*_1_ complex revealed a supernumerary subunit, which was named subunit IV (SIV) (14, 25–28). The gene that encodes SIV was later found outside of the main *fbcFBC* operon that encodes the catalytic subunits and was named *fbc*Q (29). *fbcQ* encodes a 14.4-kDa, 124-amino acid protein that shares no homology to supernumerary subunits of cyt *bc*_1_ complexes from other species or the similarly named subunit IV (PetD) of the cyt *b*_6_*f* complex. Through biochemical characterization and deletion of *fbcQ*, SIV was shown to be required for photosynthetic growth. However, since SIV is not required in aerobically grown cells, it appears to be unnecessary for the operation of cyt *bc*_1_ complexes during respiratory electron transfer (30). SIV is predicted to consist of an ~10-kDa soluble domain on the cytoplasmic face of the complex and a C-terminal TMH that is suspected to bind quinone (29, 31–33). The absence of SIV results in lowered activity and increased superoxide production, and the complex becomes less stable following detergent treatment, suggesting that SIV is required to produce a stable and fully active complex during photosynthetic growth (30, 34). Several studies showed that the SIV TMH provides the majority of its function and that specific residues in the helix are essential (35–37). Despite this wealth of biochemical data, the structure and function of SIV remain elusive due to its dissociation during purification, and it is therefore absent from existing crystal structures of the *R. sphaeroides* cyt *bc*_1_ complex (38, 39).

In this study, we applied our recently published approach utilizing styrene maleic acid (SMA) copolymer in place of detergents to achieve solubilization of cyt *bc*_1_ from the chromatophore membranes of photosynthetically grown *R. sphaeroides* (40); SMA extracts membrane protein complexes directly from the native membrane, enclosing them in nanodiscs (41–43). This strategy shows great promise for high-resolution structural determination of membrane proteins (44, 45), and in the case of cyt *bc*_1_ and cyt *b*_6_*f* complexes it preserves the local membrane environment (40). Following successful purification of active complex, we used cryogenic electron microscopy (cryo-EM) to determine the structure of the four-subunit *R. sphaeroides* cyt *bc*_1_ at 2.9 Å resolution, revealing the position of the TMH of SIV and providing insights into its function. We also resolve a natively bound quinone at the Q_o_ site and alternative conformations of the Rieske subunits, providing information on structural changes during the Q-cycle. Accompanying biochemical comparisons with the complex lacking SIV suggest a role for this component in stabilizing and enhancing uniquinol:cytochrome *c* oxidoreductase activity.

## Results

### Purification of Cyt *bc*_1_ in Native Nanodiscs.

The cyt *bc*_1_ complex sits in a lipid-rich environment within photosynthetic membranes (4, 40), adjacent to RC-light-harvesting complex 1 (RC-LH1) core complexes (46). We previously showed that SMA preferentially extracts the cyt *bc*_1_ complex from its lipid-rich surroundings within chromatophore membranes, whereas the protein-rich domains containing RC-LH1 and light-harvesting complex 2 (LH2) complexes resist the action of SMA (40). Despite low yields of His_10_-tagged cyt *bc*_1_ purified by nickel affinity chromatography, we demonstrated that the cyt *bc*_1_ dimer copurified with ~56 annular phospholipids, one ubiquinone and SIV, providing a promising strategy for structural and biochemical characterization of the cyt *bc*_1_ complex in a near-native lipid environment (40), which is imperative given that early work on the *R. sphaeroides* cyt *bc*_1_ complex revealed its sensitivity to removal of lipids (25).

To increase the yields of cyt *bc*_1_ in our preparations, we made several alterations to our previous purification protocol. First, we truncated the C terminus of the zinc transporter ZnuC, which was identified as a major contaminant in our previous work (40), by inserting a premature stop codon into the gene. This protein contains eight His residues within its C-terminal 15 amino acids, which we suspect act as a natural His-tag. Second, we commonly observe that SMA weakens the association of the His-tag with the Ni-NTA resin, so after binding, we washed the nickel column with a lower imidazole concentration to prevent undesired elution of cyt *bc*_1_. Finally, we applied the complexes eluted from the Ni-NTA column to a continuous sucrose density gradient and performed an ultracentrifugation step to separate the cyt *bc*_1_ complexes from contaminating RC-LH1 and LH2, damaged cyt *bc*_1_, and other minor contaminants prior to further purification and buffer exchange by size-exclusion chromatography.

**Fig. 1.**
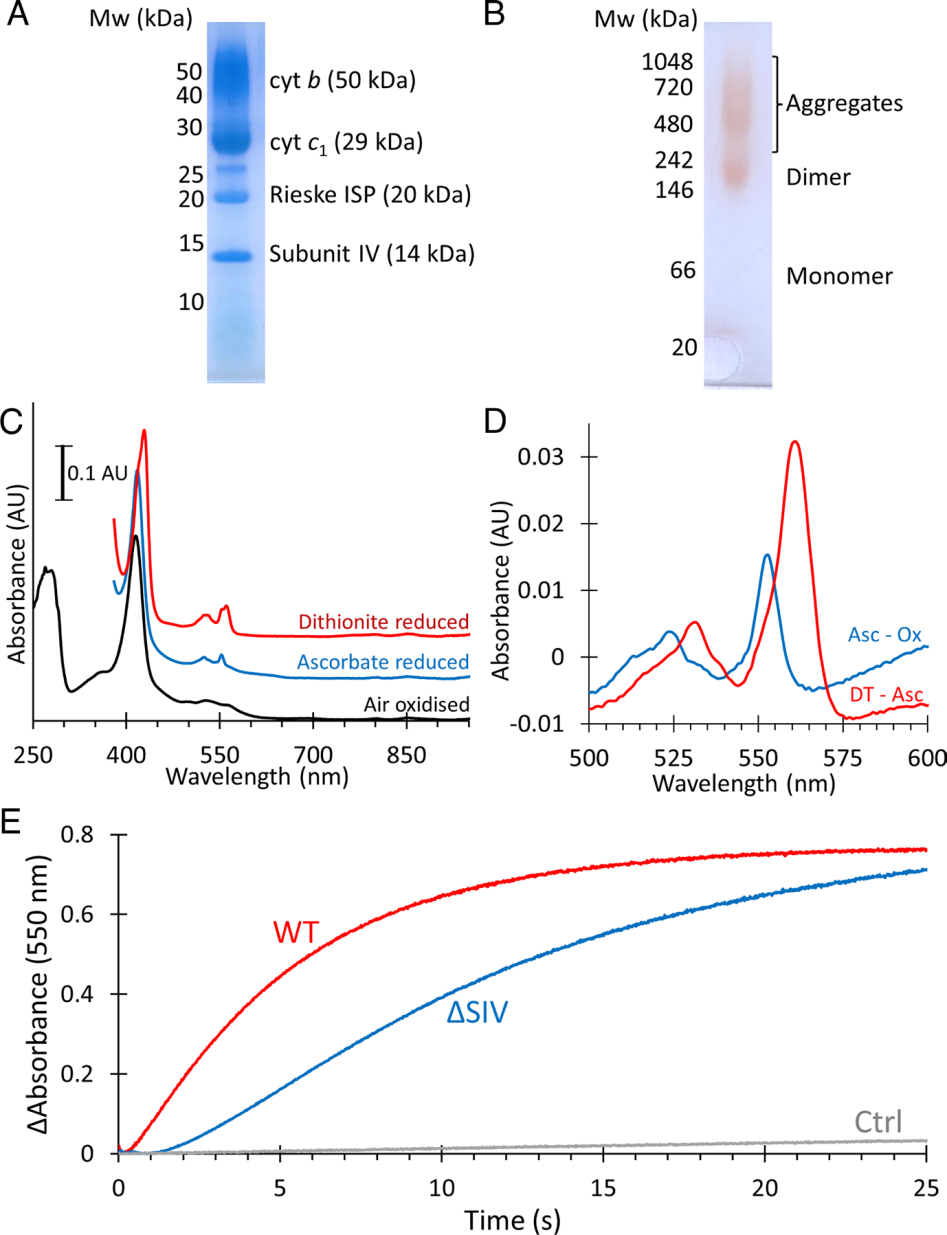
Purification and characterization of cyt *bc*_1_. (*A*) Coomassie-stained SDS–PAGE of purified cyt *bc*_1_ complexes. Each band is labeled with its identity and predicted mass. (*B*) CN-PAGE of purified cyt *bc*_1_ complexes with the oligomeric state indicated next to the bands. (*C*) UV/Vis/NIR spectra of cyt *bc*_1_ complexes as prepared (Air oxidized) and following the addition of sodium D-ascorbate to reduce *c* hemes, and sodium dithionite to reduce all hemes. (*D*) Ascorbate reduced minus ferricyanide oxidized (Asc – Ox), and dithionite reduced minus ascorbate reduced (DT – Asc) spectra used for determination of *c* and *b* heme concentrations, respectively. (*E*) Activity of purified WT (red) and ΔSIV (blue) complexes, monitored by following the absorption of cyt *c*_2_ at 550 nm. Reactions contained 500 nM cyt *bc*_1_, 50 µM cyt *c*_2_, and 250 µM decylubiquinol (solid lines). The control (Ctrl) reaction performed in the absence of cyt *bc*_1_ shows low spontaneous cyt *c* reduction (gray).

Analysis by sodium dodecyl sulfate–polyacrylamide gel electrophoresis (SDS–PAGE) shows that the cyt *bc*_1_-nanodisc complexes were highly pure, with four major bands corresponding to the three catalytic subunits and SIV (Fig. 1*A*). The presence of all four polypeptides was confirmed by mass spectrometry, with minor contamination, mainly by various other membrane proteins (*SI Appendix*, Table S1). Further analysis of the peptides revealed that SIV is complete, or subject to only limited N-terminal processing (*SI Appendix*, Fig. S1). Clear-native (CN)-PAGE (Fig. 1*B*) showed that the complex was mostly dimeric, with some higher order aggregates and a negligible amount of monomeric complex. UV/Vis/NIR spectra of oxidized and reduced cyt *bc*_1_ gave a *b-* to *c*-type heme ratio of 1.7:1, in good agreement with the expected 2:1 ratio (Fig. 1 *C* and *D*).

### Comparison of Cyt *bc*_1_ Activity in the Presence and Absence of SIV.

To ensure the complex was active, we performed cyt *c*_2_ reduction assays, which demonstrated the complex was functional in the presence of 250 µM decylubiquinol and 50 µM cyt *c*_2_ (Fig. 1*E*, red). To compare the activity of the four-subunit complex with one without SIV, we produced a strain where the *fbcQ* gene was deleted (hereafter ∆SIV), purified the three-subunit complex (*SI Appendix*, Fig. S2), and performed analogous cyt *c*_2_ reduction assays. The activity of the wild-type (WT) complex was 11 ± 2 µmol cyt *c*_2_ reduced/µmol cyt *bc*_1_/s. In the absence of SIV the activity decreased 63% to 4.0 ± 0.2 µmol cyt *c*_2_ reduced/µmol cyt *bc*_1_/s (Fig. 1*E*, blue), similar to the loss observed in detergent preparations (30). We note that the three-subunit complex has a weaker band corresponding to the Rieske subunit on SDS–PAGE gels, which may reflect damage during preparation or misassembly under photosynthetic growth conditions (*SI Appendix*, Fig. S2).

### Overall Architecture of the Four-Subunit Complex.

Using the purified and active four-subunit complexes, we prepared cryo-EM grids and collected 15,867 movies from which 4,060,135 particles were picked and subjected to multiple rounds of two-dimensional (2D) and three-dimensional (3D) classification. A single high-quality 3D class with 282,636 particles was refined to yield a C1 map with a global resolution of 2.9 Å. Refinement with imposed C2 symmetry gave no significant improvement in resolution, so we modeled the complex using the C1 map to reveal any asymmetrical features. Final validation statistics are displayed in *SI Appendix*, Table S2.

**Fig. 2.**
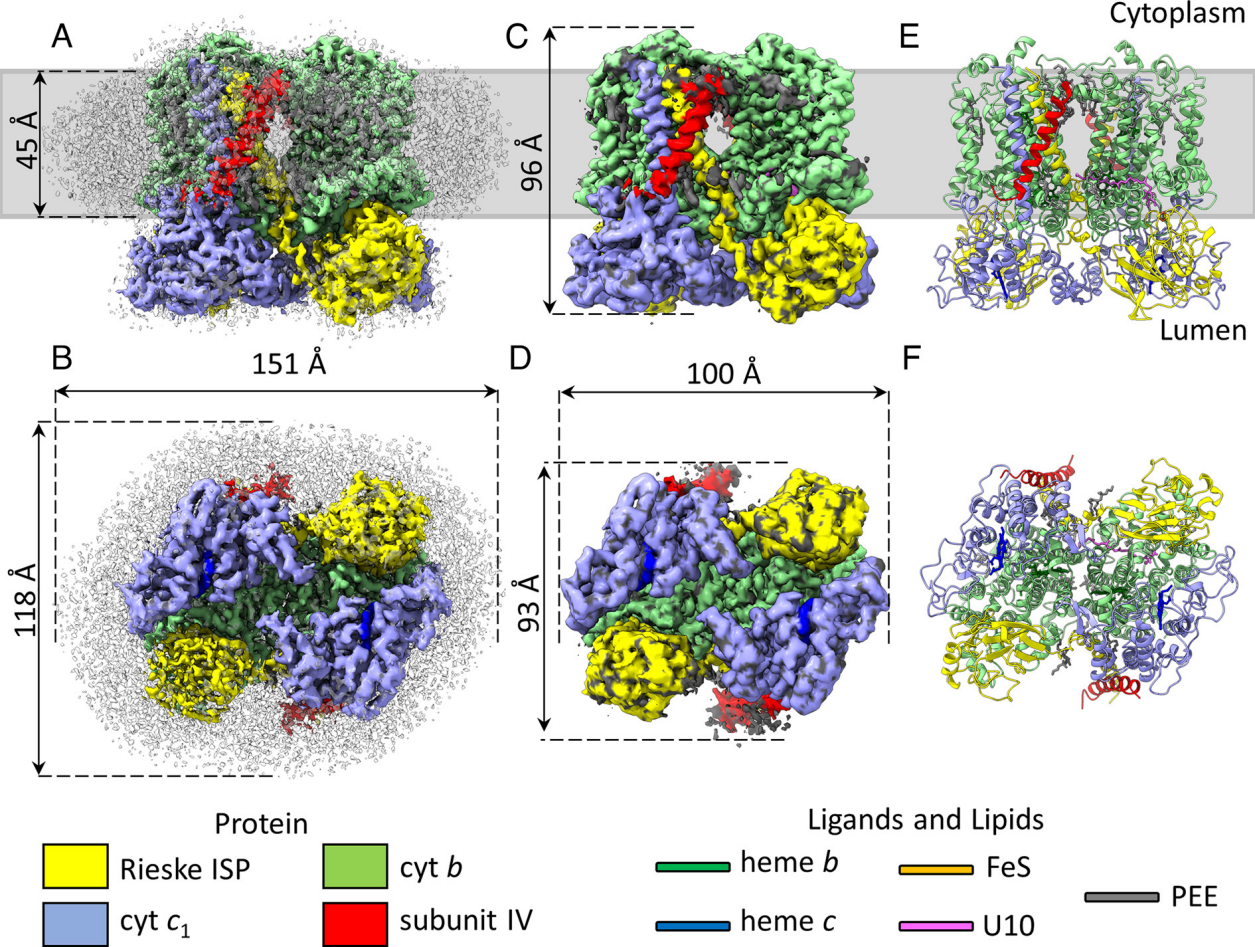
Color-coded cryo-EM map and refined model for the *R. sphaeroides* four-subunit cyt *bc*_1_ complex. (*A* and *B*) Postprocessed map viewed in the plane of the membrane (*A*) and from the lumenal face (*B*). (*C* and *D*) The map after local sharpening, which removed the disordered density of the nanodisc and improved interpretability for low-resolution portions of the map. (*E* and *F*) The refined model with the protein in cartoon representation, and ligands and lipids in stick representation. The gray bar in panels (*A*, *C*, and *E*) indicates the approximate position of the membrane. U10 = ubiquinone-10 and PEE = 1,2-dioleoyl-sn-glycero-3-phosphoethanolamine.

Fig. 2 *A*–*D* shows the cryo-EM map with colored regions corresponding to the resolved proteins. The dimensions of the dimeric complex in the membrane plane are 100 Å × 93 Å with a height of 96 Å; the complex is surrounded by a 151 Å × 118 Å × 45 Å belt of disordered density corresponding to the SMA-stabilized lipid nanodisc. There are eight TMHs and a *b*_L_ and *b*_H_ heme for each cyt *b* subunit. The *c* subunits each bind a *c*-type heme within a lumenal soluble domain, which is anchored via a single TMH. The two Rieske iron–sulfur protein (ISP) subunits each consist of one TMH associated with one half of the monomer, and a soluble head domain binding a FeS cluster that interacts with the opposing monomer on the lumenal side of the complex. In the center of the complex is an intermonomer cavity, which is largely empty. Docking an existing crystal structure of the three-subunit *R. sphaeroides* cyt *bc*_1_ complex [PDB ID:2QJP (38)] into the map produced a good agreement between the catalytic subunits of the two structures. The remaining unmodeled regions of the complex revealed a TMH on each side of the complex that, considering fit to density and physical environment, matched to residues 79-107 of SIV (Fig. 2, red). These residues correspond to the predicted transmembrane region with the expected topology, with the N terminus on the cytoplasmic side and the C terminus on the lumenal side (29, 31–33). We also observed 12 ordered lipid molecules, tentatively assigned as 1,2-dioleoyl-*sn*-glycero-3-phosphoethanolamine (PEE), and a ubiquinone-10 molecule bound in the Q_o_ site of one of the cyt *b* subunits (Fig. 2, gray and pink).

The local resolution of the map was variable with a range of ~2.7 to 4.1 Å. The highest resolution region was for the membrane-embedded cyt *b* subunits, while the soluble head domains of the Rieske subunits and the cytoplasmic region of SIV were at the lowest resolution (*SI Appendix*, Fig. S3), suggesting that these domains may be flexible. It should be noted that we chose not to add inhibitors to our complex in order to retain native quinones and permit the complexes to explore a range of active conformations. To aid interpretation of the map, we applied local sharpening after initial building using LocScale (47), shown in Fig. 2 *C* and *D*, and used this map to further refine the final model shown in Fig. 2 *E* and *F*.

### The Structure of Subunit IV.

**Fig. 3.**
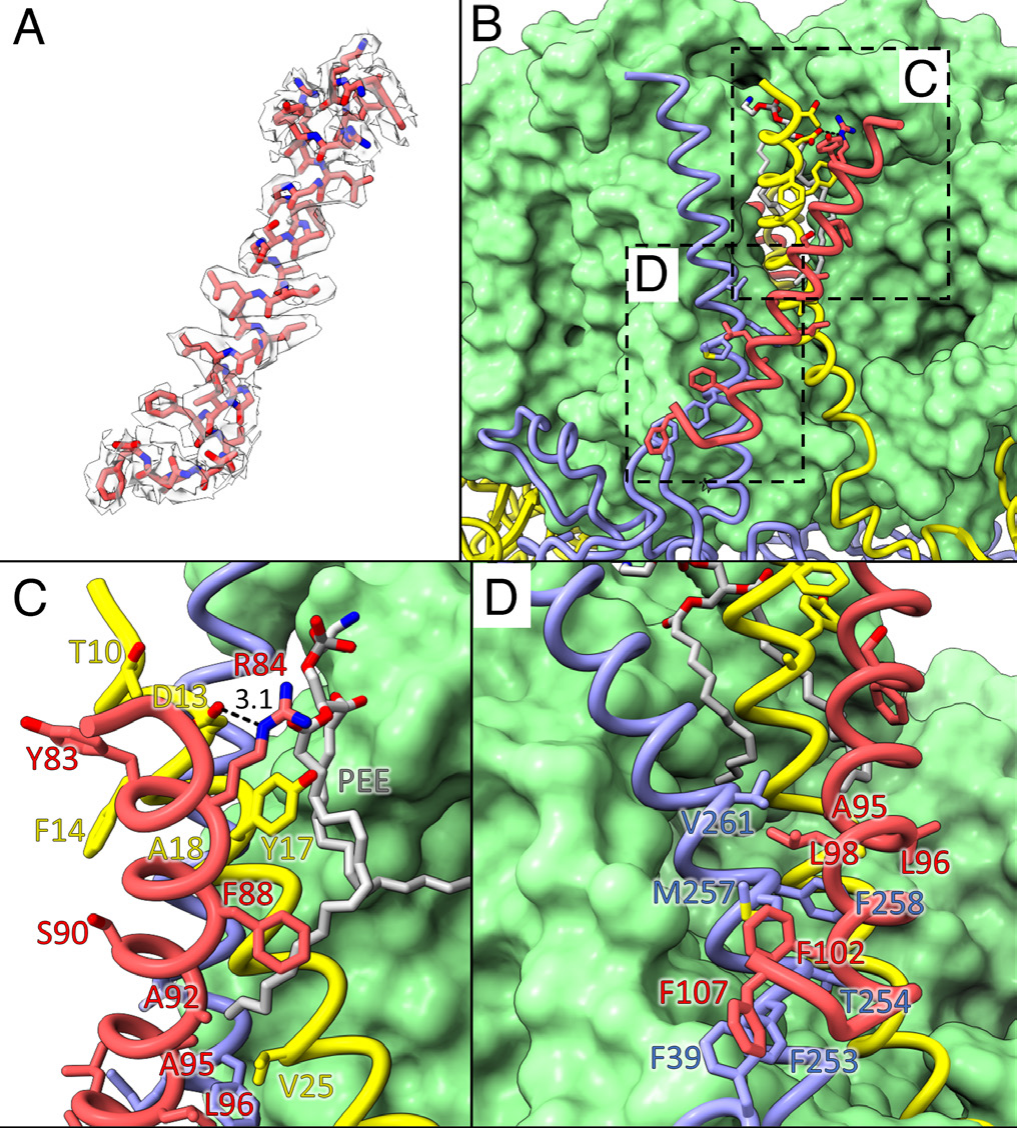
Structure of the resolved region of SIV and its interactions with the cyt *bc*_1_ complex. (*A*) Model of residues 79 to 107 of subunit IV (sticks) within its density after local sharpening (semi-transparent surface). (*B*) Expanded view of subunit IV (red) interacting with the Rieske (yellow) and cyt *c*_1_ (blue) subunits. Cyt *b* (green) is in surface representation, and the other subunits are shown as ribbons with interacting side chains as sticks. The dashed boxes indicate the areas enlarged in panels (*C* and *D*). (*C*) Expanded view of the cytoplasmic side of SIV with residues mediating the interaction with the Rieske subunit labeled. (*D*) Expanded view of the lumenal side of SIV with residues mediating the interaction with cyt *c*_1_ labeled.

The resolved TMH of SIV is 28 residues in length, corresponding to residues 79 to 107 of the 124-amino acid SIV sequence (Fig. 3*A*). It lies at an angle of ~20 to 25° relative to the membrane plane and interacts extensively with the TMHs of the cyt *c*_1_ and Rieske subunits along most of its inward-facing side (Figs. 2 and 3*B*). Most of the interactions are hydrophobic contacts within the lipid bilayer, with residues 83 to 96 packing against residues 10 to 25 of the Rieske subunit, and residues 95 to 102 packing against residues 253 to 261 of the cyt *c*_1_ subunit. On the lumenal side, there is an additional interaction between Phe107 of SIV and Phe39 within the cyt *c*_1_ head domain (Fig. 3*D*). On the cytoplasmic surface of the complex Arg84 of SIV forms a hydrogen bond with Asp13 of the Rieske subunit (Fig. 3*C*). SIV also makes hydrophobic interactions with the tail of a resolved lipid via the side chains of Phe88 and Ala92 (Fig. 3*C*).

Unexpectedly, no density for the N-terminal 78 residues, corresponding to a molecular mass of ~9.6 kDa, was observed in our refined maps. This region of SIV is predicted to be a small soluble domain on the cytoplasmic side of the membrane. SDS–PAGE suggests SIV has a molecular mass of ~15 kDa, indicating that the N-terminal region is intact in our preparation (Fig. 2*A*), and mass spectrometry shows that peptides corresponding to the unresolved region are present (*SI Appendix*, Fig. S1). We note that the cytoplasmic end of the SIV TMHs has a relatively low local resolution (*SI Appendix*, Fig. S3), and that the backbone extends away from the rest of the complex (Fig. 3 *B* and *C*). Attempts to resolve density for the N-terminal domain of SIV from the cryo-EM data by focused refinement were unsuccessful. We attempted to model the structure of SIV using AlphaFold (48), which predicted a range of conformations for the soluble domain and could not correctly position SIV within the remainder of the complex. Taken together, these data suggest that the SIV N-terminal domain is highly flexible and does not adopt a constant, resolvable conformation with respect to the remainder of the cyt *bc*_1_ complex.

### Structurally Resolved Lipids.

**Fig. 4.**
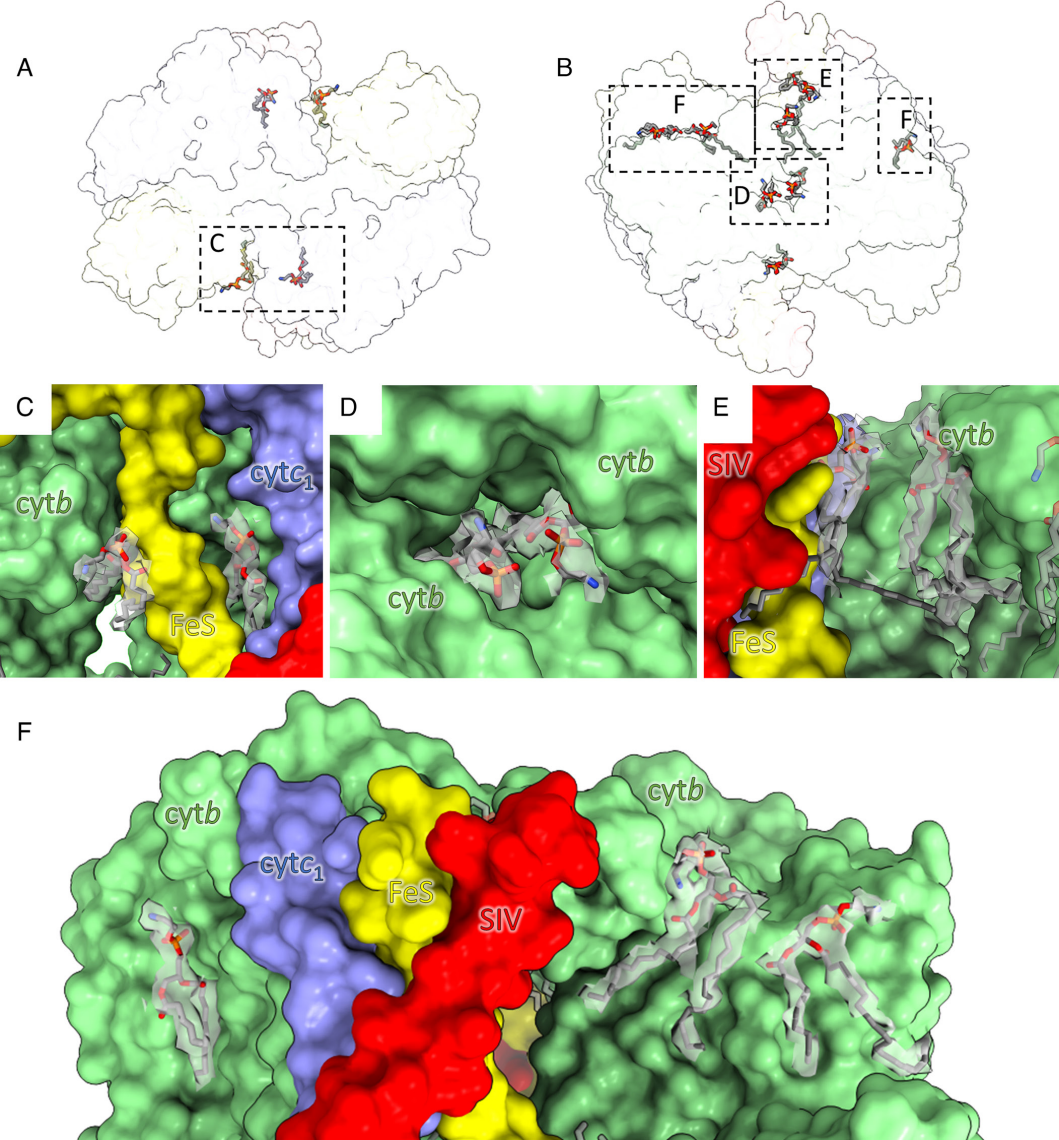
Resolved lipids in the cyt *bc*_1_ complex. (*A*) Cross-section of the complex displayed as a semi-transparent surface viewed from the lumenal side of the complex with lipids shown in stick representation. (*B*) Semi-transparent surface and lipids viewed from the cytoplasmic side of the complex. (*C*) Close-up view of one pair of lumenal lipids. The protein is shown in surface representation with each subunit labeled, and the lipids are shown in stick representation within their density, shown as a gray semi-transparent surface at threshold 0.012. (*D*) Expanded view of the cytoplasmic lipids at the center of the complex with density at threshold 0.007. (*E*) Expanded view of the cytoplasmic lipids in the central cavity at a threshold of 0.007. (*F*) Zoomed-in view of lipids on the cytoplasmic face of the complex with density at threshold 0.011. Color coding as in Fig. 2: SIV, red; cyt *c*_1_, blue; Rieske ISP, yellow; cyt *b*, green.

Following detergent-free extraction of cyt *bc*_1_ from the native membrane with SMA, we aimed to resolve the lipids that copurified with the complex. Twelve lipids were modeled into the map (Fig. 4), all of which were identified by their characteristic fork-shaped density. We were unable to unambiguously model the head groups, so tentatively assigned each as PEE and truncated the lipid tails as necessary to fit the density.

Four lipids were resolved on the lumenal side of the complex in a symmetrical arrangement where two lipids are bound within clefts on either side of the Rieske TMH, below the flexible linker that connects it to the head domain. One of these lipids interacts with the Rieske TMH and the other with the cyt *b* subunit that interacts with the Rieske head domain. The other lipid is inserted in a pore between the Rieske TMH, cyt *c*_1_, and cyt *b* subunits in the opposing monomer. The lipid head group is closely associated with the protein, and the tails extend toward the intermonomer cavity where they become disordered and can no longer be resolved (Fig. 4 *A*, *C*).

The remaining eight lipids were modeled on the cytoplasmic side of the complex, and their locations can be divided into three groups (Fig. 4*B*). There are two lipids bound within the central cavity of the complex that interact with both cyt *b* subunits (Fig. 4*D*). Two more lipids were resolved within a cavity between cyt *b* and the TMHs of SIV, the Rieske subunit and cyt *c*_1_ monomer. One of these lipids interacts with the Rieske, cyt *c*_1_, and SIV TMHs, and the second is bound to cyt *b* with one tail extending toward the Q_i_ site and the other toward the central cavity (Fig. 4*E*). On the opposite side of the complex, only the lipid interacting with cyt *b* was resolved (Fig. 4*B*). We note that density was apparent in the symmetrical location, but this was not of sufficient quality to allow confident assignment and was left unmodeled. Three additional lipids were modeled on the outside of the complex associated with the cyt *b* subunits on only one monomer; density features could not be assigned with any confidence on the other side of the complex (Fig. 4*F*).

### Ubiquinone Bound to the Q_o_ Site and Conformational Changes in the Rieske Head Domain.

**Fig. 5.**
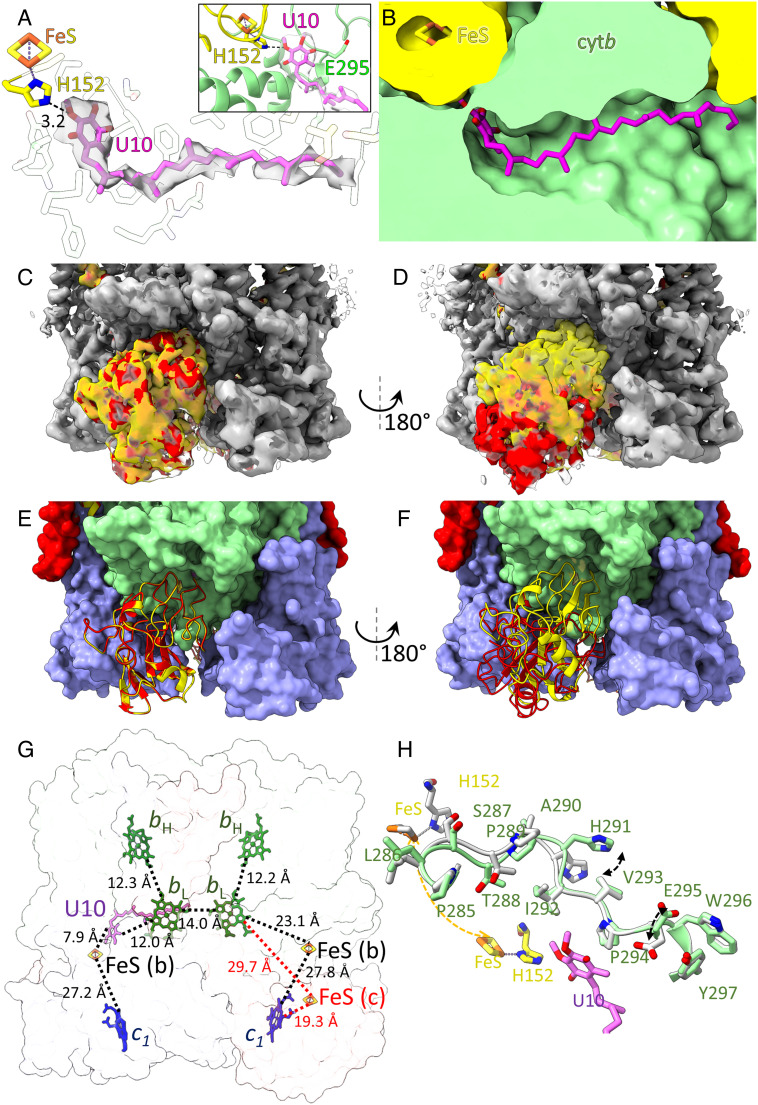
Resolved quinone at the Q_o_ site and Rieske subunit dynamics. (*A*) Ubiquinone-10 (U10, magenta) within its density from the unsharpened map at threshold 0.07 (semi-transparent gray surface). The hydrogen bond to Rieske His152 is shown with a black dashed line and labeled with its distance. Other interacting residues are shown with semi-transparent sticks. The inset shows the proximity of U10 to Glu295, which is too distant for hydrogen bonding. (*B*) A cut-away surface view showing the ubiquinone binding pocket. (*C*) Overlaid locally sharpened consensus and focused-class maps. The static cyt *b* and cyt *c*_1_ subunits and SIV are shown in gray, and the Rieske subunits are shown in semi-transparent yellow and solid red for the consensus map and the focused-class map, respectively. (*D*) The maps shown in panel *C* rotated 180° to show the mobile Rieske subunit. (*E*) Refined model of the focused-class with all subunits in surface representation except the near Rieske subunits, which are both resolved in the *b*-position, shown as cartoons. (*F*) The model in *E* rotated 180 degrees. The yellow Rieske subunit is resolved in the *b*-position, and the red subunit has rotated into *c*-position. (*G*) Hemes, FeS clusters, and U10 shown in stick representation. Closest edge-to-edge distances are shown in dashed black lines for the consensus structure, and red lines for the FeS cluster when the Rieske subunit is in the *c*-position. (*H*). Overlays of cyt *b* residues 285 to 297 in the apo (gray) and holo (color) Q_o_ sites from the focused and consensus classes, respectively. A movie showing morphs between the two structures in this figure is provided in the Movie S1.

Following modeling of the protein, cofactors, and lipids, an area of unassigned density that resembles a quinone headgroup with an isoprene tail was apparent (Fig. 5*A*). After fitting a ubiquinone-10 molecule and truncating the isoprene tail to fit the density, we found that the quinone/quinol was bound within the Q_o_ site. For a thorough interpretation of the structure, it is important to consider the quinone species bound to the Q_o_ site, which cannot be determined from density at the 2.9 Å resolution of the map. The samples were prepared predominantly in darkness to minimize quinol formation by the RCs. Following separation from the other complexes, the cyt *bc*_1_ complexes had sufficient time to equilibrate to their resting state. Therefore, it is most likely that a quinone is bound at the Q_o_ site and His152 is protonated. This is consistent with previous work, where in the absence of inhibitors and excess quinone a gx = 1.800 signal is observed in electron paramagnetic resonance, which is assigned to the Q.ISPH from of the Q_o_ site (49). However, it is important to note that we cannot rule out unintended alterations of the redox state during cryo-EM grid preparation. The head group of the quinone is near the Rieske subunit, forming a hydrogen bond (3.2 Å) between the quinone methoxy oxygen and His152, which chelates the FeS cluster. As this oxygen has no exchangeable H, this is unlikely to be a redox active conformation. The redox active oxygen is 4.5 Å away from the His152 nitrogen, which is too far for proton transfer. Therefore, the quinone conformation is likely to represent a snapshot of quinone approach to, or egress from, the Q_o_ site rather than the enzyme-substrate complex. The position of the head group is close to that of the Q_o_ site inhibitor stigmatellin bound to the *R. sphaeroides* complex lacking SIV [PDB ID: 2QJP (38)] (*SI Appendix*, Fig. S4*A*). However, unlike for stigmatellin, Glu295 within the conserved PEWY motif is too distant to form a hydrogen bond (5.45 Å) because the sidechain has rotated away from the quinone head group (Fig. 5*A*, *Inset*). It has been suggested that while Glu295 is important for stigmatellin binding, movement of semiquinone during the catalytic cycle and proton release (50–54), it is not required to bind quinone, consistent with the Q_o_ quinone in our structure. The ubiquinone in our structure is bound slightly deeper within the Q_o_ site than in analogous structures of mitochondrial complex III with the Q_o_ site occupied [PDB ID:6Q9E (55), PDB ID:7RJA (56); *SI Appendix*, Fig. S4*B*], and in a similar position to menaquinone, in close proximity to the FeS cluster, in menaquinone-binding complexes (57). Therefore, the present structure may add a further snapshot of conformations representing the approach and binding of ubiquinol to the Q_o_ site within the cyt *bc*_1_ complex. There is also a series of hydrophobic interactions along the length of the molecule in the cyt *b* subunit. These residues (cyt *b* Ile91, Met140, Ala141, Phe144, Met145, Gly158, Ile162, Val161, Trp179, Leu180, Leu197, Pro294, Phe298, Phe301 Tyr302, Leu305, Met336; opposing cyt *b* Ile63, Val64, Met67; Rieske Leu34, Ile35, Met38 and Cys151) line a hydrophobic channel that guides the quinol from within the membrane to the Q_o_ site at the membrane surface (Fig. 5 *A* and *B*). We note that other densities were present, both within the opposing Q_o_ site and within both Q_i_ sites, but they could not be reliably assigned and were left unmodeled.

The relatively low local resolution of the Rieske subunits may be indicative of dynamics in this subunit, so we performed focused classification to resolve distinct conformations. We began by creating a soft mask around the soluble domains of the Rieske subunits, then performing 3D classification without alignment. One of the three classes, comprising 72,118 particles, showed an alternative conformation for one Rieske head domain. Following unmasked refinement of this class without alignment, we were able to rigid-body dock the Rieske head domain in a new orientation, rebuild the loop that connects it to the TMH, locally sharpen the map using LocScale (47), and perform molecular dynamics flexible fitting with ISOLDE (58). The resulting map (resolution at FSC 0.5 = 3.75 Å) is shown overlaid with the consensus map in Fig. 5 *C* and *D* and in Movie S1. One of the Rieske head domains has rotated, moving from a position where it is docked with the cyt *b* subunit (the *b*-position) to one where it points toward the cyt *c*_1_ subunit (the *c*-position) (Fig. 5*D* and Movie S1). In contrast, both cyt *b* and cyt *c*_1_ subunits, both SIVs, and the other Rieske subunit overlay almost exactly (Fig. 5 *C* and *D* and Movie S1). Comparison of the models reveals that the Rieske head domain has rotated 52° relative to the subunit in the *b*-position owing to a rearrangement of the flexible linker between the head domain and the TMH. The rotation angle is slightly more than that observed in the *R. capsulatus* complex determined by cryo-EM (59), and slightly less than in the crystal structures of the *R. sphaeroides* and chicken cyt *bc*_1_ complexes (12, 52, 60). A rotation of 56° was observed in molecular dynamics simulations (61), indicating that we have captured an intermediate state with the Rieske subunit almost fully shifted toward the *c*-position. The model also reveals that the stationary Rieske domain interacts with the bound Q_o_ quinone, while the mobile Rieske domain is on the side of the complex containing an unoccupied Q_o_ site (Fig. 5*C*–*G*). This suggests that the interactions between His152 and the bound quinone (Fig. 5*A*) bias the Rieske subunit toward the cyt *b* proximal conformation, whereas when the Q_o_ site is in the apo state, the Riseke subunit is free to adopt a wider range of conformations.

Fig. 5*G* and the Movie S1 show overlays of the cofactors and quinone in both structures with closest edge-to-edge distances labeled. All distances except those between the FeS cluster on the mobile side and its neighboring heme *b*_L_ and heme *c*_1_ are identical. In the *b* position, the FeS cluster is 27.8 Å from heme *c*_1_ and 23.1 Å from heme *b*_L_. Following rotation into the *c* position the distance to heme *c*_1_ decreases to 19.3 Å and the distance to heme *b*_L_ increases to 29.7 Å. On the opposing, static side of the complex, the quinone is located between the FeS cluster and heme *b*_L_. The edge-to-edge distance to the FeS cluster is 7.9 Å and the distance to heme *b*_L_ is 12.0 Å. The remaining cofactors are found in the same arrangement observed in other cyt *bc*_1_ structures and facilitate rapid electron transport via the low-potential chain toward the Q_i_ site (62), and intermonomer electron transfer via heme *b*_L_ (63, 64). Fig. 5*H* shows alignments of residues 185 to 297 of the cyt *b* subunit between the quinone-bound Q_o_ site in the consensus structure, and the unoccupied site in the focussed refined structure. There is a clear rearrangement of the cyt *b* lumenal loop (residues 285 to 296) with a notable shift in the position of His291 and of Glu295, which has rotated into the apo Q_o_ site.

## Discussion

### Interactions of Subunit IV with Other Components of the Cyt *bc*_1_ Complex.

The fourth subunit of the *R. sphaeroides* cyt *bc*_1_ has long been known to be necessary for its optimal function, but the structure has remained elusive owing to its absence from crystal structures (38, 65). By combining purification of the complex in native nanodiscs with cryo-EM, we have resolved the structure of the TMH of SIV. The topology of SIV is in good agreement with previous studies, which demonstrated that the soluble N-terminal domain is located within the cytoplasm and the C-terminal region comprises a single TMH flanked by a small soluble lumenal domain (29, 31–33). In contrast to previous proposals that SIV interacts with the cyt *b* subunit, we show that its TMH lies across those of the Rieske and cyt *c*_1_ subunits. Structural alignments with mitochondrial cyt *bc*_1_ complexes reveal that SIV occupies a position similar to that adopted by the 7.2-kDa supernumerary subunit in the mitochondrial cyt *bc*_1_ complex (yeast QCR9 or mammalian UQCR10) (PDB ID:1BGY (13), *SI Appendix*, Fig. S5), which is required for correct incorporation of the Rieske subunit and to stabilize the dimeric complex (66–69). Intriguingly, the acidic C-terminal region of yeast QCR9 is not needed for function, meaning its TMH appears be the major functional domain (66). This suggests that the TMH of SIV may serve to stabilize the complex, strengthening the interactions with the Rieske TMH and, consequently, the dimer interface. This is in keeping with reports that cyt *bc*_1_ complexes from *R. sphaeroides* mutants lacking SIV are more labile following solubilization with dodecyl maltoside detergent (30). SIV and QCR9/UQCR10 do not share significant sequence similarity, suggesting they have evolved independently to fulfil their roles. We note that structural alignments of our four-subunit cyt *bc*_1_ complex with the plant and cyanobacterial cyt *b*_6_*f* complexes [PDB IDs 6RQF and 7R0W (70, 71)] reveal the absence of an equivalent of the *R. sphaeroides* SIV in cyt *b*_6_*f*.

Usui and Yu previously indicated that the TMH of SIV interacted with quinones using radiolabeled quinone derivatives (29). Chen et al. showed that mutation of Trp79, which is the first residue we resolved on the cytoplasmic face of the membrane, impaired activity of the complex by ~75%, and raised the *K*_m_ for quinol fourfold (35). This residue is distant from the quinone/quinol binding sites and lies at the entrance to the intermonomer cavity, interacting with lipids that bridge to the Q_i_ site. In combination, these previous studies and the present structure indicate that Trp79 may be involved in transient interactions that guide quinones diffusing from the quinone pool to the Q_i_ binding pocket.

The primary structure of SIV has been the subject of intense previous characterization. Wu and Niederman performed partial digestion of the four-subunit cyt *bc*_1_ and liberated a 9- to 10-kDa fragment of SIV, which corresponds to the 9.6-kDa mass of the unresolved region. Intriguingly they found that the ubiquinol-cyt *c* reductase activity of the complex was not diminished following digestion (31). Deletion of *fbc*Q from the genome results in cyt *bc*_1_ complexes with activity decreased by 75% and a 4.3-fold increase in *K*_m_ for quinol (30). It was also shown that SIV was specifically produced during photosynthetic growth and that its deletion did not impede growth under aerobic conditions, suggesting SIV is specifically incorporated into cyt *bc*_1_ complexes in chromatophores (30). Chen et al. later showed that activity could be restored with a plasmid-encoded copy of SIV and that residues 1 to 5 and 113 to 124 (a predicted small C-terminal soluble region), which were not resolved in our structure, were not required. Deletion of other residues in the soluble domain resulted in incorrect or no incorporation of SIV, suggesting that the N-terminal domain is required for the assembly and stability of SIV in vivo (37). In a later study, Chen et al. purified the three-subunit complex from a SIV-deficient strain and reconstituted it with SIV produced recombinantly in *Escherichia coli*, which restored almost all ubiquinol-cyt *c* reductase activity at a stoichiometric ratio. Tso et al. performed reconstitutions with truncated SIV peptides and showed that the transmembrane region was absolutely required for restoration of activity (36). Loss of the first 41 amino acids did not impede activity, while ~50% recovery was achieved with fragments containing the TMH between residues 77 to 109 (37). Further investigation by Tso et al. showed that the YRYR motif (residues 81 to 84) was essential, two residues of which (Tyr83 and Arg84) make protein–protein interactions in our structure (Fig. 3). Together, these studies indicate that interactions of the transmembrane region of SIV give rise to most of its function. The susceptibility of the N-terminal domain to proteases along with the finding that much of it is not required for normal activity suggests that it makes minimal, if any, interactions with the rest of the complex in mature cyt *bc*_1_, explaining why it is flexible and unresolved in our maps. Additional functions cannot be discounted for the soluble domain, for example interacting with other proteins or complexes. One example could be the universal stress protein-1, which enhances cyt *bc*_1_ activity and reduces superoxide production in the presence of SIV (72).

### The Role of Subunit IV in Increasing the Rate of ATP Production of a Photosynthetic Vesicle.

By combining our present structure with the results of previous studies, we can propose a function for SIV. By binding to the TMHs of the Rieske subunit and cyt *c*_1_, SIV enhances the stability of the complex, and also increases its activity (29) (Fig. 1*E*) and lowers the production of damaging superoxide (34). Under aerobic growth, particularly under oxygen-limiting conditions, SIV is not required, either because the activity of the three-subunit cyt *bc*_1_ is sufficient to sustain respiratory electron transport, or because interactions with cyt *c* oxidase and cyt *c*_y_ to form supercomplexes provide their own set of stabilizing interactions (59, 73). However, under photosynthetic conditions, the RC can reduce quinol and oxidize cyt *c*_2_ at a higher rate than during respiratory electron flow, even at relatively low light intensities (4, 5, 9). The assembly of a dimeric RC-LH1 core complex in *R. sphaeroides* (74–76) elevates the efficiency of energy trapping (77), placing more importance on the downstream oxidation of quinols generated by the RC. By incorporating the supernumerary SIV, which raises the maximum turnover rate of the cyt *bc*_1_ complex up to threefold, and by positioning the cyt *bc*_1_ complex in a lipid-rich domain adjacent to the RC (4, 5, 40, 46), *R. sphaeroides* has maximized this step in the overall conversion of absorbed light energy to ATP. However, the turnover of the enhanced four-subunit cyt *bc*_1_ is still rate-limiting for the overall process of ATP synthesis when viewed from the perspective of the whole chromatophore vesicle (4, 5, 9). A photosynthetic chromatophore comprises on average 67 LH2 complexes, 11 RC–LH1 dimers, 2 RC–LH1 monomers, 4 cyt *bc*_1_ dimers, and 2 ATP synthases (4). It has been suggested that the prevailing stoichiometry of approximately three RC-LH1 dimers to one cyt *bc*_1_ dimer limits pmf generation by cyt *bc*_1_ complexes, thereby offering protection against damage to the membrane vesicle by over-acidification. Another beneficial aspect of this stoichiometry is the overcapacity of ATP synthase activity, which is more than threefold higher than that required to consume the protons generated by the cyt *bc*_1_ complexes (10). In summary, the activity of the four-subunit cyt *bc*_1_ is optimized at the level of single complexes, but is also necessarily suboptimal at the level of a whole chromatophore vesicle. Thus, because cyt *bc*_1_ activity is the kinetic bottleneck for converting light energy to ATP, the acquisition of SIV has raised ATP production by a photosynthetic vesicle almost threefold. The only other possibility of achieving a similar outcome for a vesicle would be to overcome the bottleneck by assembling threefold more cyt *bc*_1_ complexes that lack SIV, which would be substantially more costly for the cell.

### Binding of a Native Quinone within the Q_o_ Site of the Cyt *bc*_1_ Complex.

The RMSD of our consensus structure to the three-subunit complex with bound stigmatellin and antimycin (PDB ID:2QJP) was 0.460 Å, showing that transition to a detergent environment does not induce major conformational rearrangements within the complex. One notable difference is a small shift of the N-terminal region of the Rieske TMH, which is likely to accommodate the binding of SIV, and a small difference in the position of the Rieske hydrophilic domains (*SI Appendix*, Fig. S6). Because of the enhanced stability afforded by the native nanodisc assembly, and the ability to sample native conformational flexibility in cryo-EM datasets, we chose to exclude inhibitors from our preparations, and to process the data without imposing symmetry. This resulted in an asymmetric model where one of the Q_o_ sites had clear density for a bound quinone, while the other had weak density that could not be unambiguously assigned. A hydrogen bond between the Q_o_ quinone and His152 in the Rieske head domain locks it in the *b*-position with the FeS cluster close to the Q_o_ site. This interaction has previously been described in crystal structures with stigmatellin bound at Q_o_ (*SI Appendix*, Fig. S4) (12, 38, 52). Focused refinement revealed alternative conformations in the Rieske subunit proximal to the apo Q_o_ site, whereby rotation of the Rieske head domain to the *c*-position moves the FeS cluster closer to the *c*-heme within cyt *c*_1_ (Fig. 5*G*). This conformation is similar to that observed by Steimle et al. for the *R. capsulatus* cyt *bc*_1_ complex determined by cryo-EM (59), and cyt *bc*_1_ complexes from various other species with a range of inhibitors bound (12, 60). A shift to the *c*-position after reduction of the FeS cluster following electron transfer from quinol significantly accelerates forward electron transfer to heme *c*_1_ while preventing the unwanted back reaction in which an electron returns to the quinone in the Q_o_ site or to heme *b*_L_, ensuring high efficiency of the bifurcated electron transfer that underpins the Q-cycle (52, 61, 78).

Structural alignments of the apo and holo Q_o_ sites revealed a conformational shift in the loop of the cyt *b* subunit spanning residues 285 to 297, that includes the PEWY motif. The most notable differences are the rotation of His291, which involves a small rearrangement of the backbone to accommodate the new conformation, and a rotation of Glu295 into the apo Q_o_ site. These changes are similar to those observed in crystal structures of the *R. sphaeroides* complex with either stigmatellin or azoxystrobin bound (60). A rotation of the PEWY glutamate was also observed in the chicken structure with either stigmatellin or myxothiozol bound (Glu272), but the rest of the loop is largely similar between the two structures (50, 52). Previous studies have shown that both Glu295 and His291 are necessary for proton release during oxidation of quinol at the Q_o_ site in bacterial complexes (50, 79). Therefore, the conformational differences we observe between the holo and apo sides of our structure may represent conformational changes involved in proton channelling during turnover. The observation of conformational rearrangements highlights the benefits of combining cryo-EM and native nanodiscs, and of not imposing symmetry on the maps for observing dynamics and quinone/quinol binding in these complexes. These approaches will be applicable to other cyt *bc*_1_ and cyt *b*_6_*f* complexes, and many other membrane-intrinsic protein complexes.

### Concluding Remarks.

In summary, the structure of the four-subunit cyt *bc*_1_ complex of *R. sphaeroides* resolves many previously open questions surrounding the structure and function of SIV and reveals a correlation between quinone/quinol binding at the Q_o_ site and conformational changes in the Rieske head domain. Our structure provides intriguing insights into how and why supernumerary subunits of cyt *bc*_1_ have evolved, and in particular how the acquisition of a single protein component of a rate-limiting complex can elevate the photosynthetic production of ATP.

## Materials and Methods

### Generation of Strains.

Genomic modifications were made using the pK18mob*sacB* plasmid as previously described (80). The 3′ end of *znuC* (rsp_3568) was modified by addition of two premature stop codons seven residues before the C terminus. This was achieved by overlap extension PCR with primers to amplify the mutated end of the gene with ~400-bp upstream and downstream flanking genomic DNA (*SI Appendix*, Table S3). The sequence was ligated into pK18mob*sacB* using restriction enzymes EcoRI and HindIII, and the resulting plasmid was conjugated into *R. sphaeroides* harboring a His_10_-tagged cyt *c*_1_ (4) using *E. coli* S17-1. Correctly modified strains were isolated following sequential selection and counter selection steps with kanamycin (30 µg/mL) and sucrose (10% w/w), respectively, and were verified by PCR and automated Sanger sequencing (Eurofins).

*The fbcQ* (rsp_2687) gene was deleted from the strain containing the truncated ZnuC modification by amplifying ~400-bp regions upstream and downstream of *fbcQ* and joining them by overlap extension PCR, yielding a genomic sequence lacking the SIV coding region (*SI Appendix*, Table S3). The resulting PCR fragment was cloned into pK18mob*sacB* and used to delete the *fbcQ* gene as described above.

### Preparation of cyt *bc*_1_ in Native Nanodiscs.

Purification of cyt *bc*_1_ was performed by adapting our previous method (40) to enhance both purity and yield of the complex. *R. sphaeroides* cells were grown to stationary phase under photosynthetic conditions in 9 L M22 media (81) under ~50 µmol m^−2^ s^−1^ illumination from 70 W Phillips Halogen Classic bulbs. This corresponds to an optical density at 650 nm of ~5 for the strains with SIV and ~3 for those lacking SIV due to their impaired photosynthetic growth. Cells were harvested by centrifugation at 4,000 RCF for 30 min at 4 °C then resuspended in 30 mL working buffer (20 mM Tris-HCl pH 8 containing 200 mM NaCl). Following addition of a few crystals of DNaseI and lysozyme, cells were broken via two passes through a cell disruptor (Constant Systems, UK) at 20,000 PSI followed by removal of unbroken cells and insoluble debris by centrifugation at 25,000 RCF for 15 min at 4 °C. To separate the membranes, the supernatant was centrifuged at 150,000 RCF for 2 h at 4 °C, and the soluble fraction was discarded. The pigmented membrane pellets were resuspended in 20 mL working buffer and homogenized. Cyt *bc*_1_ was solubilized by diluting membranes to an absorbance at 850 nm of 50 in working buffer containing 1.5 % w/w SMA [prepared as described previously (82)] and incubating at room temperature for 1 h in the dark with gentle stirring. Insoluble material was removed by centrifugation at 150,000 RCF for 1 h at 4 °C, and the soluble fraction was diluted twofold prior to binding to a 50 mL Ni-NTA column preequilibrated in five column volumes (CVs) of working buffer by recycling for 16 h at a flow rate of 5 mL min^−1^. The column was washed with ten CVs of working buffer containing 10 mM imidazole then cyt *bc*_1_ was eluted over a linear gradient of 10 to 250 mM imidazole over six CVs collecting 5 mL fractions. UV/Vis/NIR spectra of pigmented fractions were collected between 350 and 1,000 nm, and those with a Soret band between 413 and 415 nm and a ratio of absorbance between 415 and 850 nm above 1 were concentrated to ~1 mL using a 100,000 MWCO Amicon centrifugal filter (Merck). Cyt *bc*_1_ was further enriched by applying 0.5 mL to a 11 mL 10 to 15% w/w sucrose gradient in working buffer and centrifuging at 100,000 RCF for 16 h at 4 °C. The pigmented bands at ~12% sucrose were fractionated, spectra were collected between 250 and 1,000 nm, and those with a Soret band at 414 to 415 nm and a ratio of absorbance between 415 and 850 nm above ten were pooled and concentrated to 1 mL. Cyt *bc*_1_ was injected onto a HiPrep™ 16/60 Sephacryl® S-300 HR size exclusion column (Cytiva) preequilibrated with two CVs of working buffer and eluted over 1.5 CVs at 0.5 mL/min collecting 2.5 mL fractions. Fractions with a Soret band at 415 nm and a ratio of absorbance at 415 nm to 280 nm of 1.15 to 1.25 were retained. The absorbance in the 800 to 900 nm range corresponding to RC-LH1 and LH2 was negligible in the final preparation and could be disregarded. The samples were concentrated to a *b*-heme concentration of 15 µM and used immediately for cryo-EM grid preparation, or frozen using liquid nitrogen in working buffer containing 20% v/v glycerol and stored at −80 °C until required.

### UV/Vis/NIR Spectroscopy.

Spectra were collected between 250 and 1,000 nm on a Cary 60 spectrophotometer. Spectra of pure cyt *bc*_1_ were collected immediately following dilution in working buffer. Next a few grains of potassium ferricyanide were added followed by a few grains of sodium D-ascorbate and finally a few grains of sodium dithionite, collecting new spectra ~30 s after the addition of each reagent. The *c* heme concentrations were determined using the ascorbate reduced minus ferricyanide oxidized spectrum with an extinction coefficient of 20 mM^−1^ for the 551 nm to 542 nm absorbance difference (83). The *b*-heme concentration was determined using the dithionite reduced minus ascorbate reduced spectra with an extinction coefficient of 28 mM^−1^ for abs560–abs574 (84). For all assays, the cyt *bc*_1_ concentration was taken as [*b*-heme]/2.

### Activity Assays.

Oxidized cyt *c*_2_ was prepared by mixing equine cyt *c*_2_ (Merck) with a few grains of potassium ferricyanide, followed by removal of ferricyanide on a PD-10 desalting column (Cytiva). Removal of oxidant was confirmed by UV/Vis spectroscopy, and the cyt *c*_2_ concentration was determined by reducing a small amount with sodium D-ascorbate and using an extinction coefficient of 29.5 mM^−1^ at 550 nm. Decylubiquinol was prepared by reduction with sodium dithionite in 50% v/v ethanol and extracted by phase-separation with hexane as previously described for decylplastoquinone (71). Decylubiquinol was dissolved in a minimum volume of DMSO, and the concentration was determined using an extinction coefficient of 4 mM^−1^ at 290 nm in ethanol (85).

Activity assays were performed using an OLIS RSM1000 spectrophotometer equipped with a USA-SF stopped flow cell operating in rapid-scanning mode at 25 °C with a scan rate of 31 s^−1^ between 350 and 700 nm with 300 line mm^−1^/500-nm blaze gratings, a 1.24-mm entrance slit, and 0.6-mm exit slit. Solution A contained 625 nM cyt *bc*_1_ and 62.5 µM oxidized cyt *c*_2_. Solution B contained 1.25 mM decylubiquinol. The solutions were mixed in a 4:1 ratio to give final concentrations of 500 nM cyt *bc*_1_, 50 µM cyt *c*_2_ and 250 µM decylubiquinol. Initial linear rates were determined using OLIS Global Works and Microsoft Excel software packages with a cyt *c*_2_ red-ox extinction coefficient of 21 mM^−1^ at 550 nm.

### Cryo-EM Grid Preparation.

Quantifoil 1.2/1.3 300 mesh Cu grids (EM Resolutions Ltd.) were glow discharged at a current of 10 mA for 12 s under a partial atmosphere of air using a Cressington 208 glow discharge unit. A 5 µL volume of 15 µM cyt *bc*_1_ was applied to the carbon-coated side of the grid and incubated at 18 °C with 80% relative humidity for 30 s, blotted for 4 s, and then plunge frozen in liquid ethane at −178 °C. Grids were screened using a Talos Arctica FEG TEM operating at an accelerating voltage of 200 kV equipped with Falcon III direct electron detector at 78,000 × magnification and a defocus of −3 µm. Grids with uniform thin ice and good particle density within the holes were selected for data collection.

### Cryo-EM Data Collection.

A total of 15,867 movies were collected on an FEI Titan Krios equipped with a Gatan K3 direct electron detector operating in super resolution mode with an energy filter slit width of 20 eV and an accelerating voltage of 300 kV. The nominal magnification was 130,000×, yielding an imaging resolution of 0.651 Å per physical pixel at the detector. Movies were collected over a 1.13 s exposure and fractionated into 40 frames with a dose of 1 e^−^ Å^−2^ frame^−1^ for a total dose of 40 e^−^ Å^−2^. The applied defocus ranged between −0.8 and −2.0 µm in 0.3 µm steps.

### Cryo-EM Data Processing.

Data processing was performed using Relion 3.1 (86) unless otherwise stated. Movies were motion corrected using Relion’s implementation of MotionCorr2 (87) with dose weighting using 5 × 5 patches and a B factor of 150, followed by CTF estimation using CTFFind 4.1.14 (88) within Relion. 4,060,135 particles were picked using crYOLO 1.7.5 (89) using a model trained with 10 motion corrected micrographs. Particles were extracted in 360-pixel boxes (downscaled to 120 pixels) and subjected to five rounds of reference-free 2D classification (T = 1.3, 540 classes in the final round), selecting those classes that have features corresponding to a nanodisc encapsulated complex of ~16 × 12 × 10 nm after each round. The final selected 2D classes contained 1,452,666 particles (35.8%). Next, a de novo initial model was generated using the stochastic gradient descent method with three classes, and the best class was used as a reference for 3D classification following removal of strong signal at the mask edge in UCSF ChimeraX 1.3 (90). The particles were subjected to two rounds of 3D classification (T = 4, 10 classes in round 2), yielding a single class with clear secondary structure features comprised of 282,636 particles (7.0%). Particles from this class were reextracted in 396-pixel boxes downscaled to 220 pixels (corresponding to a pixel size of 1.1718 Å at the specimen level) and subjected to multiple rounds of 3D refinement with two rounds of per-particle CTF refinement (fitting magnification, defocus, astigmatism, B-factor, phase-shift, beam tilt, trefoil and fourth order aberrations) and one round of per-particle motion correction. The final map after masking solvent (extending by 3 pixels and applying a 3-pixel soft edge) and postprocessing had a global resolution of 2.9 Å (FSC = 0.143). Selected 2D and 3D classes are shown in *SI Appendix*, Fig. S7, and FSC curves are shown in *SI Appendix*, Fig. S8.

### Model Building and Refinement.

An initial model was generated by downloading an existing *R. sphaeroides* cyt *bc*_1_ structure from the PDB (PDB ID: 2QJP (38)) and removing inhibitors, waters, detergents, and ions. The model was docked into the map using the “Fit in map” tool in ChimeraX 1.3 (90) and manually refined in Coot v0.9.6 (91). The model was subtracted from the map to visualize unmodeled regions, which revealed two clear alpha-helical segments on each side of the complex. These were modeled by fitting a polyalanine helix into the map, then mutating the side chains to give best-fit to the density while considering the local environment using the SIV sequence as a reference, then manually building missing regions. Next, lipids and quinones were modeled into the remaining density and truncated to give a reasonable fit. The complete model was further optimized using the ISOLDE 1.2 plugin for ChimeraX (58) followed by final real-space refinement in Phenix v1.19.2 (92). Following local sharpening of the map in LocScale (47) the model was further refined using Coot, ISOLDE, and Phenix. Validation statistics are displayed in *SI Appendix*, Table S1.

### Focused 3D Classification and Refinement.

To analyze the refined particles for movements in the Rieske head domain, a soft mask was generated in ChimeraX 1.3. A surface was generated for residues 45 to 187, and a mask was generated on the grid of the postprocessed map extending by six pixels and padding by three pixels using the “volume onesmask” command. The mask was used for 3D classification on the refined particles with the complete map as a reference with the following settings: T = 160, 3 classes, 25 iterations and image alignment disabled. The three classes contained 72,118 (24.6% of refined particles), 79,575 (27.2%), and 140,943 (48.2%) particles. Particles from class 1, which displayed a distinct conformation for one Rieske subunit, were selected and subjected to 3D refinement without alignment followed by masking and postprocessing. The other two classes were near identical to the consensus map and not processed further. The Rieske head domain from the consensus model was reoriented to give best fit to the new map, the linker to the TMH was rebuilt in Coot, and the model was subjected to the same refinement procedure using Coot, ISOLDE, and Phenix described for the consensus model. Because disabling alignment is problematic for gold standard FSC calculations, we quote the resolution at an FSC of 0.5 (*SI Appendix*, Fig. S9 for the FSC curves), which was 3.75 Å for class 1. Validation statistics are displayed in *SI Appendix*, Table S1.

### Confirmation of Cyt *bc*_1_ Subunit Composition by Mass Spectrometry.

Proteins were extracted by precipitation from purified cyt *bc*_1_ complex and digested with pepsin as previously described (93). The resultant peptide fragments were analyzed by nanoflow liquid chromatography–mass spectrometry (94) using a 75-min reverse-phase gradient for peptide separation. Protein identification was as previously described (93).

## Supplementary Material

Appendix 01 (PDF)

Movie S1.

## Data Availability

The structures and EM maps have been deposited in the protein data bank (PDB) and electron microscopy data bank (EMDB) with the following accession IDs: Consensus refinement with the Rieske domain in the *b*-position: PDB ID: 8ASI (95), EMDB: EMD-15616 (96). Focussed class with the Riseke domains in the *b*- and *c*- positions: PDB ID: 8ASJ (97), EMDB: EMD-15617 (98). The mass spectrometry proteomics data have been deposited to the ProteomeXchange Consortium via the PRIDE partner repository (http://proteomecentral.proteomexchange.org) with the dataset identifier PXD039340 (99). All study data are included in the article and/or *SI Appendix*.
