# Supplementary material for: Cryo-EM structure of the four-subunit Rhodobacter sphaeroides cytochrome bc1 complex in styrene maleic acid nanodiscs
Source: Proc Natl Acad Sci U S A. 2023 Mar 13;120(12):e2217922120. doi: 10.1073/pnas.2217922120 (PMC10041115; doi:10.1073/pnas.2217922120)
Supplement: Supplementary file 1 — Appendix 01 (PDF) [file pnas.2217922120.sapp.pdf]

PNAS

Supporting Information for

Cryo-EM structure of the four-subunit *Rhodobacter sphaeroides* cytochrome bc_1 complex in styrene maleic acid nanodiscs

David J. K. Swainsbury[1,2,*], Frederick R. Hawkings[2,3], Elizabeth C. Martin[2], Sabina Musiał[2,†], Jack H. Salisbury[2], Philip J. Jackson[2], David A. Farmer[3], Matthew P. Johnson[2], C. Alistair Siebert[3], Andrew Hitchcock[2] and C. Neil Hunter[2]

Corresponding Author: Dr David J K Swainsbury

Email: d.swainsbury@uea.ac.uk

This PDF file includes:

Tables S1 to S3

Figures S1 to S9

Table S1. Peptides detected by mass spectrometry analysis of purified four-subunit cyt bc_1 following digestion with pepsin. Orange shading highlights the four cyt bc_1 subunits resolved in the structure.

Protein	Uniprot ID	P value			Peptide spectrum matches			Sequence coverage (%)		
		Rep1	Rep2	Rep3	Rep1	Rep2	Rep3	Rep1	Rep2	Rep3
Cytochrome *b*	Q3IY10	0.000	0.000	0.000	166	171	202	55.51	56.18	56.85
Cytochrome c_1	Q3IY11	0.000	0.000	0.000	194	223	218	65.96	66.67	69.12
Ubiquinol-cytochrome *c* reductase iron-sulfur subunit	Q3IY09	0.000	0.000	0.000	21	22	24	47.06	49.73	59.36
Cytochrome bc_1 subunit IV	Q3J2Z2	0.000	0.000	0.000	11	11	15	48.39	41.94	35.48
NADH dehydrogenase subunit L	Q3J3E6	0.000	0.000	0.000	10	13	15	8.89	10.97	13.06
Uncharacterized protein	Q3J5M7	0.000	nd	nd	2	nd	nd	9.63	nd	nd
Reaction center protein M chain	Q3J1A6	0.000	0.011	0.000	3	1	3	7.79	3.25	9.74
Probable cytosol aminopeptidase	Q3J2C5	0.001	0.000	0.000	8	7	11	9.61	10.22	14.93
Uncharacterized protein	U5NRE4	nd	0.001	0.003	nd	2	1	nd	40.91	22.73
Adenylosuccinate synthetase	Q3J0Z3	0.001	nd	0.033	2	nd	1	3.95	nd	2.33
Flagellar biosynthesis protein	Q3J1Y1	0.002	nd	0.026	2	nd	2	0.99	nd	0.99
Succinate dehydrogenase flavoprotein subunit A	Q3IZ75	0.002	0.000	0.005	4	6	6	6.00	8.17	9.83
Cytochrome c_y	Q3J003	0.003	nd	nd	2	nd	nd	9.20	nd	nd
Putative tape measure protein	Q3IWX5	0.007	0.012	0.041	1	1	1	1.03	1.03	1.03
Sec-independent protein translocase protein TatA	Q3J3D6	0.010	0.000	0.001	2	2	2	30.00	30.00	30.00
Non-homologous end joining protein Ku	Q3J0I8	0.010	nd	nd	1	nd	nd	4.87	nd	nd
ABC oligo/dipeptide transporter, inner membrane subunit	Q3J2X9	0.021	0.000	nd	5	4	nd	5.99	5.99	nd
Reaction center protein H chain	Q3J170	0.022	0.009	nd	3	3	nd	5.77	9.23	nd
L-threonine 3-dehydrogenase	Q3J3U9	0.047	nd	nd	11	nd	nd	2.05	nd	nd
Uncharacterized protein	Q3J6L2	nd	0.005	nd	nd	1	nd	nd	1.60	nd
Arginine utilization protein RocB	Q3IZA1	nd	0.009	nd	nd	1	nd	nd	1.31	nd
Transcriptional regulator, winged helix family	Q3IWQ0	nd	0.009	nd	nd	1	nd	nd	3.18	nd
Glycosyltransferase	Q3IYT3	nd	0.019	nd	nd	2	nd	nd	1.45	nd
Uncharacterized protein	Q3IVS8	nd	0.019	nd	nd	1	nd	nd	4.11	nd
Cytochrome *c* oxidase subunit Cox1	Q3J5A7	nd	0.023	0.000	nd	2	2	nd	3.00	2.65
YkuD domain-containing protein	Q3J5N3	nd	0.028	0.041	nd	2	2	nd	3.57	3.57
Protein RdxB	P54932	nd	0.045	nd	nd	4	nd	nd	4.61	nd
H-NS histone family protein	U5NMX2	nd	0.048	0.007	nd	2	2	nd	9.78	9.78
Na^+/solute symporter	Q3J0D4	nd	0.050	nd	nd	1	nd	nd	1.65	nd
ATP synthase subunit beta 2	Q3HKH4	nd	nd	0.021	nd	nd	2	nd	nd	2.16
Ysc84 domain-containing protein	Q3J2M1	nd	nd	0.032	nd	nd	1	nd	nd	4.76
Amino acid/amide ABC transporter substrate-binding protein, HAAT family	Q3J5H5	nd	nd	0.032	nd	nd	2	nd	nd	4.60
Propionyl-CoA carboxylase regulator	Q3J4E6	nd	nd	0.038	nd	nd	3	nd	nd	3.47
Phage-related protein, putative phage tail tape measure protein, lambda family	Q3J604	nd	nd	0.043	nd	nd	4	nd	nd	1.80
UvrABC system protein B	Q3J4P6	nd	nd	0.045	nd	nd	2	nd	nd	2.06
ABC Fe^{3+}-siderophore transporter, inner membrane subunit	Q3IWR6	nd	nd	0.046	nd	nd	2	nd	nd	2.89

Table S2. Cryo-EM data collection, refinement, and validation statistics.

	Cyt bc_1 consensus (b-b) (EMD-15616) (PDB-8ASI)	Cyt bc_1 focussed refine (c-b) (EMD-15617) (PDB-8ASJ)
**Data collection and processing**		
Magnification	130 000 x	-
Electron exposure (e$^-$/Å^2)	40	-
Defocus range	-0.8 to -2.0 µm	-
Pixel size (Å)	0.651	-
Symmetry imposed	C1	C1
Initial particle images (no.)	4 060 135	-
Final particle images (no.)	282 636 (7%)	72 118
Initial model used (PDB code)	RELION *de novo* model	-
Map resolution (Å)	2.9	3.75
FSC threshold	0.143	0.500
Model resolution range (Å)	2.6 – 4	Not determined
Map sharpening *B* factor (Å^2)	-117.187 (unsharpened map)	-114.875 (unsharpened map)
**Model Composition and validation**		
Non-hydrogen atoms	14726	14726
Protein residues	1789	1789
Ligands	21	21
Bond lengths (Å)	0.004 (0)	0.003 (0)
Bond angles (°)	1.079 (12)	0.804 (22)
Validation		
MolProbity score	1.38	1.40
Clashscore	5.03	3.80
Rotamer outliers (%)	0.00	0.21
Ramachandran plot		
Favoured (%)	97.41	96.50
Allowed (%)	2.54	3.38
Outliers (%)	0.06	0.11

Table S3: Primers used for generation of strains. Restriction sites used for cloning are underlined.

Primer Name	Sequence
znuC TGATGA UF	CCGGAATTCGAGGTGGCGGACCGTCAG
znuC TGATGA F	CACGACCATACGtgatgaCACGATCATTCC
znuC TGATGA R	GGAATGATCGTGtcatcaCGTATGGTCGTG
znuC KO DR	CGGCAAGCTTGCGAGGATGTCGCCGAAGAG
RSP2687 UF	CCGGAATTCCGATCATCTCGCGCCTCAAGATGGACGCCGACATGTTCTCATTCATCGTCTATCCCATC
RSP2687 UR	GATGGGATAGACGATGAATGAGAACATGTCGGCGTC
RSP2687 DF	TTCTCATTCATCGTCTATCCCATCGAATGACCCGGGCTACAAATGAGCACGCGTCTCAAGCTTGCCG
RSP2687 DR	CGGCAAGCTTGAGACGCGTGCTCATTTGTAGC

MFSFIDDIPSFEQIKARVRDDLRKHGWEKR
WNDSRLVQKSRELLNDEELKIDPATWIWKR
MPSREEVAARRQRDFETV**WKYRYRLGGFAS**
GALLALALAGIFSTGNFGGSSDAGNRPSVV
YPIE

Figure S1. Sequence of SIV (Uniprot ID Q3J2Z2). Peptides identified by mass spectrometry of purified cyt bc_1 highlighted green and resolved residues underlined in bold text.

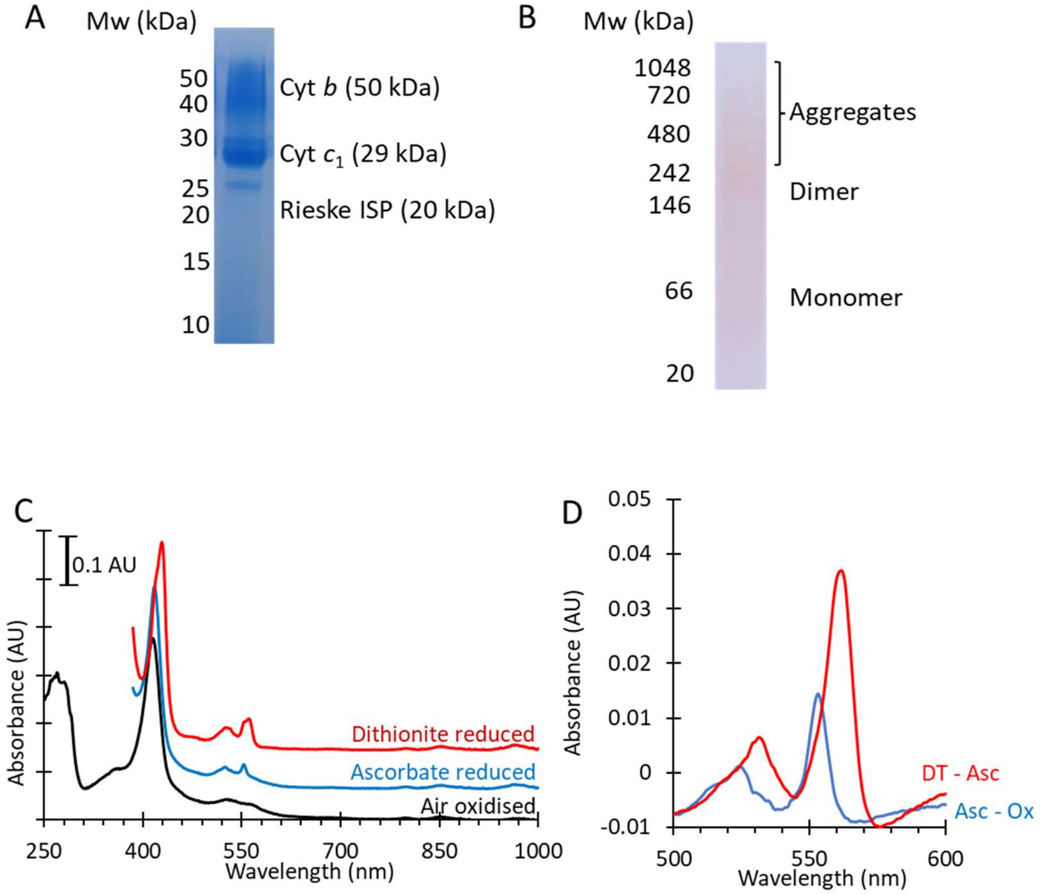

Figure S2. Gels and spectra of purified cyt bc_1 complexes from the ΔSIV background. (A) Coomassie stained SDS page gel of purified ΔSIV cyt bc_1 complexes. Each band is labelled with its identity and mass. (B) CN-PAGE gel of purified ΔSIV cyt bc_1 complexes with the oligomeric state indicated. (C) UV/Vis/NIR spectra of ΔSIV cyt bc_1 complexes as prepared (air oxidised) and following the addition of sodium ascorbate to reduce the *c* heme, and sodium dithionite to reduce all heme. (D) Ascorbate reduced minus ferricyanide oxidised (Asc – Ox), and dithionite reduced minus ascorbate reduced (DT – Asc) spectra in the 500-600 nm region used for determination of *c* and *b* heme concentrations, respectively. The heme *b* to heme *c* ratio was 1.7:1.

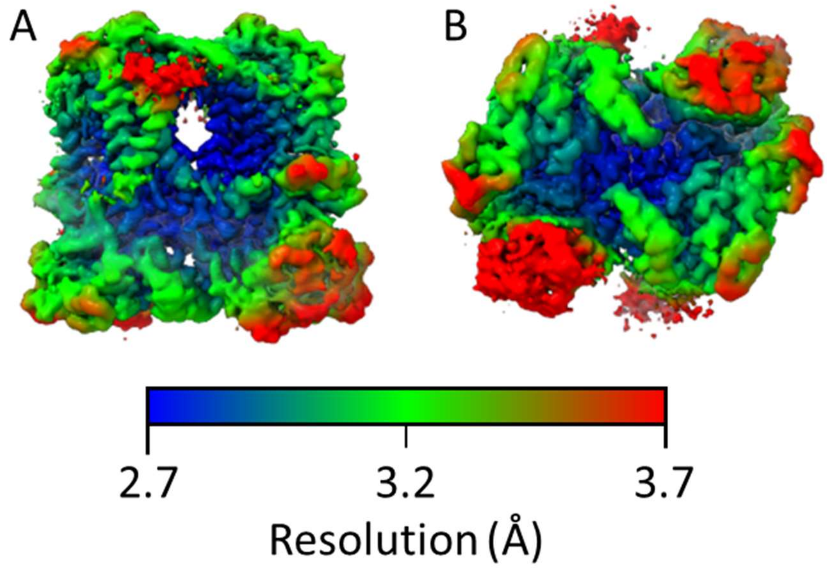

Figure S3. **Locally sharpened maps viewed in the plain of the membrane (A) and from the periplasmic face (B).** The scale bar indicates the local resolution.

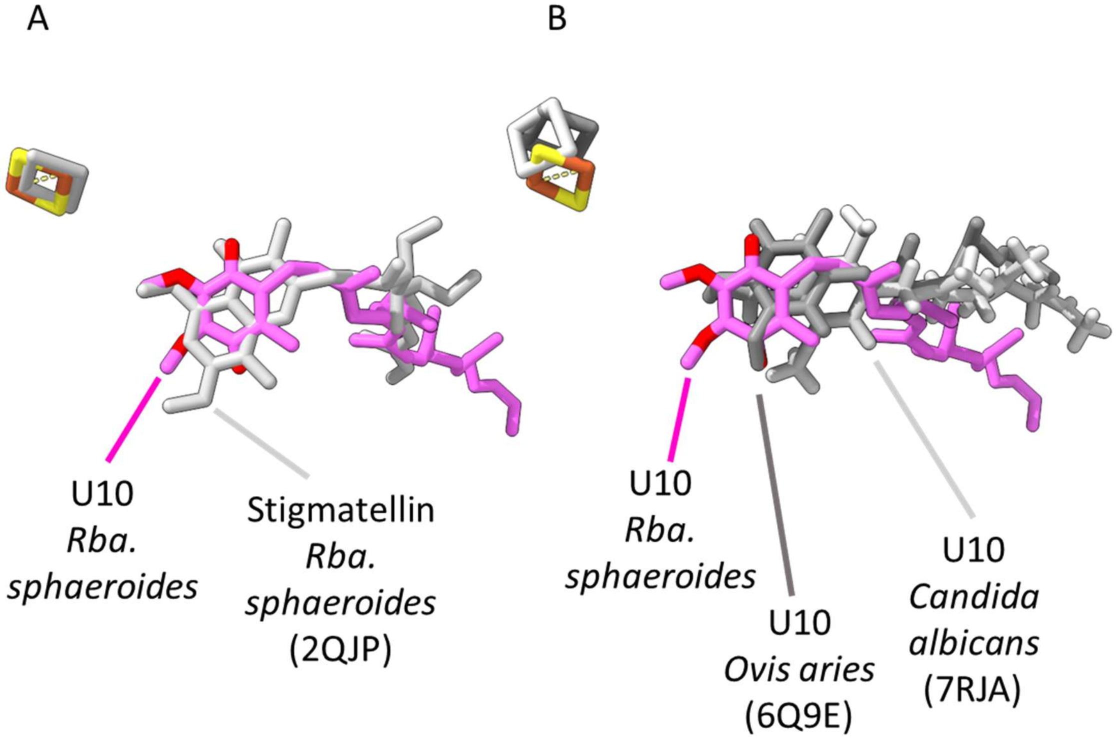

Figure S4. Comparison of the position of ubiquinone (U10) and stigmatellin in the Q_o sites of cyt *bc*$_1$ complexes. (A) Overlay of U10 in our structure with stigmatellin bound to the *Rba. sphaeroides* complex (PDB ID: 2QJP). (B) Overlay of U10 in our structure with Q_o quinones bound to complex III from *Ovis aries* (PDB ID:6Q9E, Dark grey) and *Candida albicans* (PDB ID:7RJA, light grey).

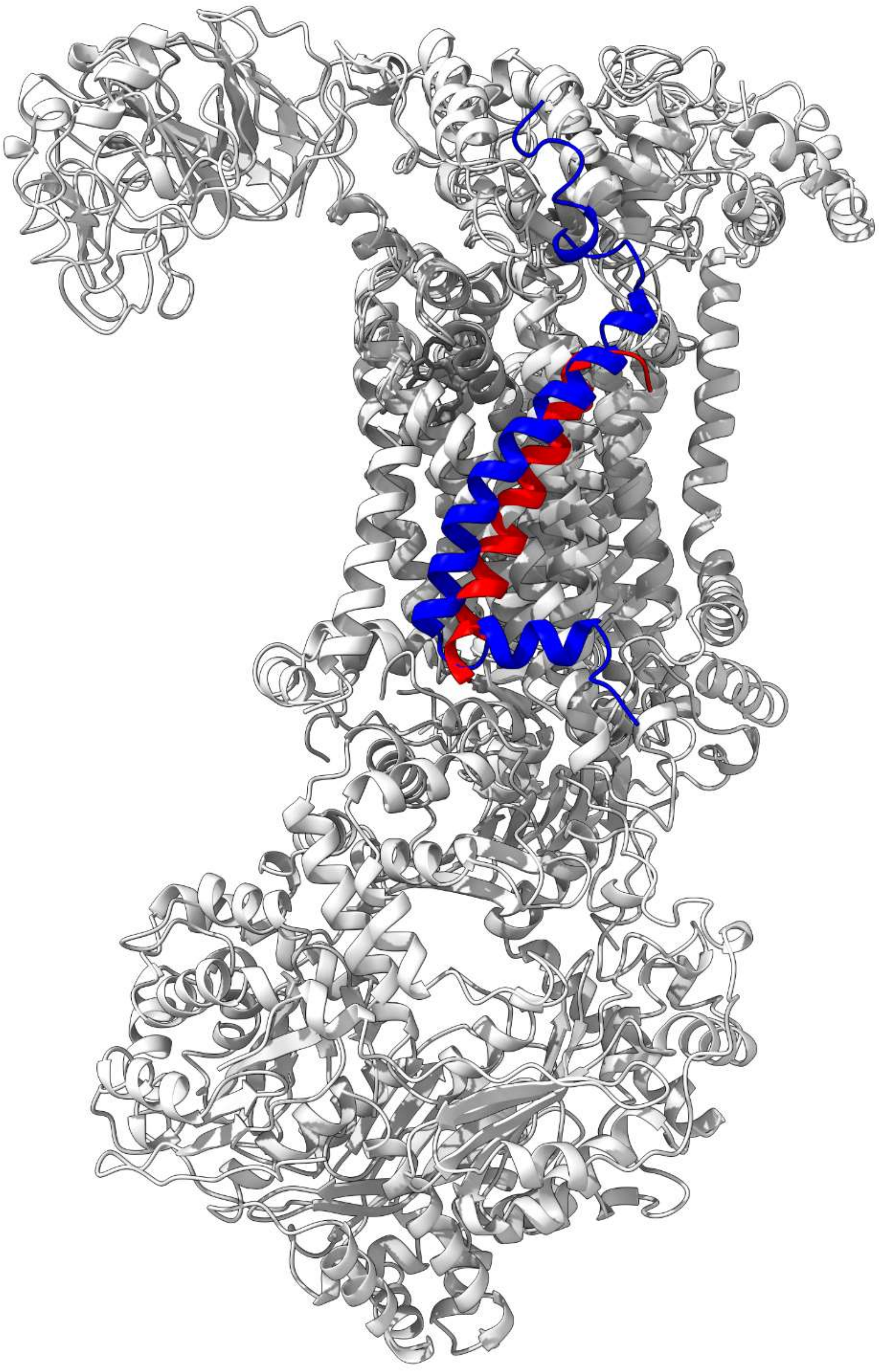

Figure S5. Comparison of the position of *Rba. sphaeroides* SIV with the UQCR10 subunit in the bovine complex. One monomer from the four subunit *Rba. sphaeroides* cyt bc_1 and the eleven-subunit bovine complex III (PDB ID: 1BE3) aligned to their catalytic subunits. Subunits for both complexes are shown in grey except *Rba. sphaeroides* SIV (red) and bovine UQCR10 (blue).

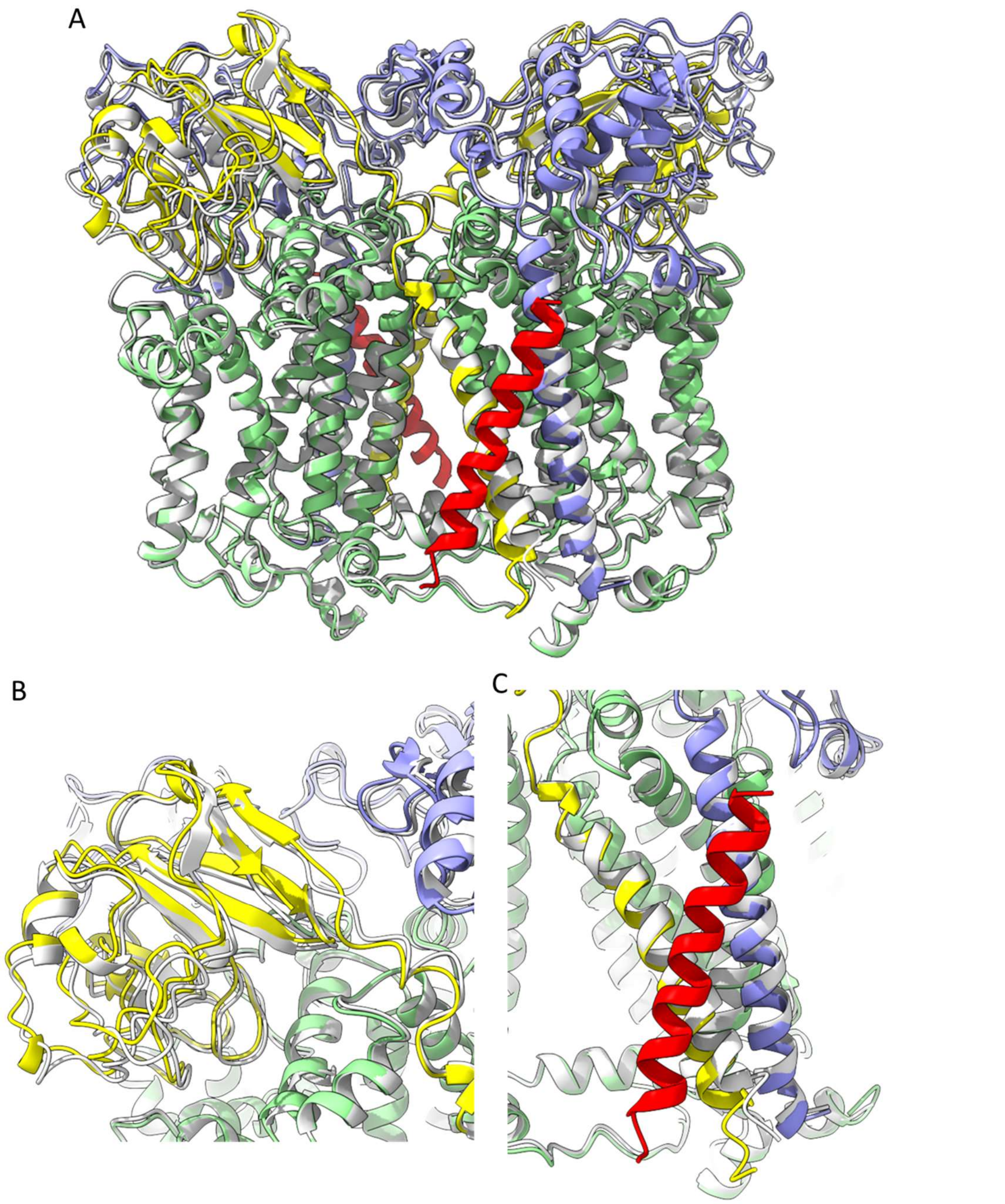

Figure S6. Alignment of the four- and three-subunit *Rba. sphaeroides* cyt bc_1 complexes. Alignment of our cryo-EM structure (coloured as in Figure 1 of the main paper) with the crystal structure of the complex lacking SIV (grey) (PDB ID: 2QJP). (A) Complete views of both complexes. (B) Zoomed in view of one Rieske head domain. (C) View of the SIV, cyt c_1 and Rieske TMHs.

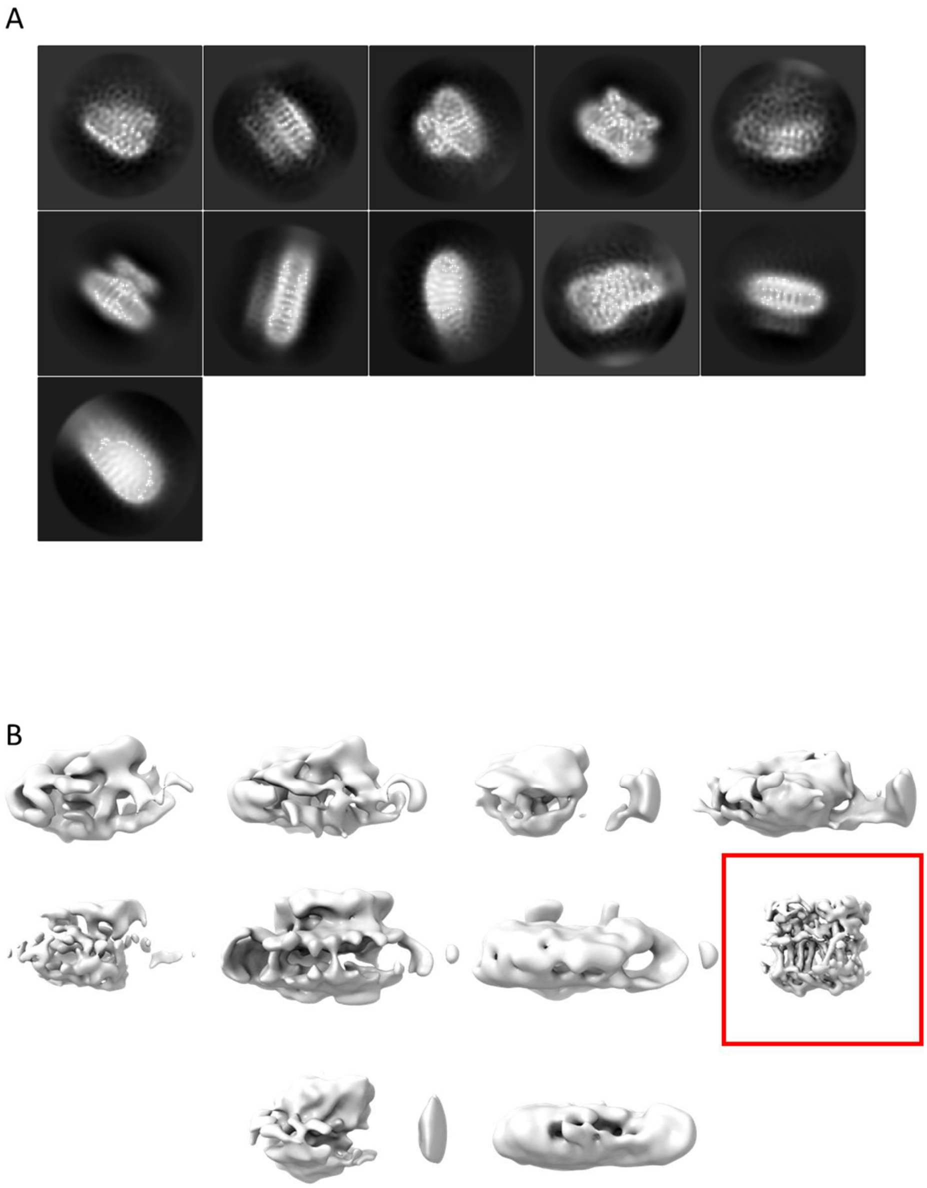

Figure S7. 2D and 3D classes from cryo-EM data processing. (A) Selected 2D classes after the fifth round of 2D classification. (B) 3D classes after two rounds of 3D classification. Class 8, indicated with a red square, was taken forward for refinement.

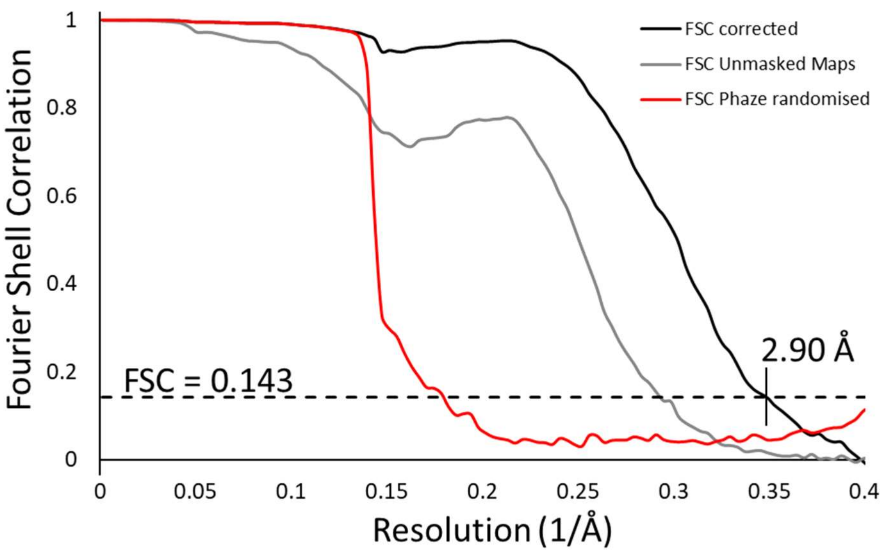

Figure S8. FSC curves for the final map. The resolution was determined with FSC = 0.143.

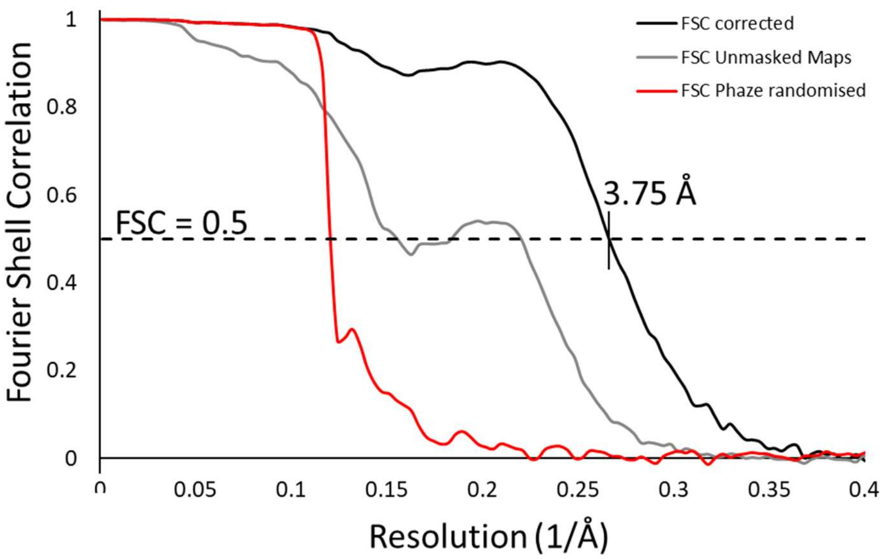

Figure S9. FSC curves for the focussed class showing an alternative conformation for the Rieske subunit. The resolution was estimated with FSC=0.5.
